# Open source surgical fracture table for digitally distributed manufacturing

**DOI:** 10.1371/journal.pone.0270328

**Published:** 2022-07-15

**Authors:** J. K. Bow, N. Gallup, S. A. Sadat, J. M. Pearce

**Affiliations:** 1 Department of Materials Science & Engineering, Michigan Technological University, Houghton, MI, United States of America; 2 Department of Biomedical Engineering and Mechanical Engineering, Michigan Technological University, Houghton, MI, United States of America; 3 Department of Electrical & Computer Engineering and Ivey School of Business, Western University, London, ON, Canada; Semnan University, ISLAMIC REPUBLIC OF IRAN

## Abstract

Roughly a third of the surgical procedures the World Bank is prioritizing as essential and cost-effective are orthopedic procedures. Yet in much of the developing world, prohibitive costs are a substantial barrier to universal access. One area where this is clear is surgical fracture tables, which generally cost >US$200,000 new. With the advent of 3-D printing, a new way to reduce medical equipment costs is to use open source hardware licensed designs to fabricate digitally-distributed manufactured medical hardware. That approach is applied here to make surgical tables more accessible. This study describes the design and manufacture of an open source surgical fracture table that uses materials that are widely available worldwide with specialty components being 3-D printed. The bill of materials and assembly instructions are detailed and the fracture table is validated to perform mechanically to specifications. Using an open source desktop RepRap-class 3-D printer, the components can be printed in a little over a week of continuous printing. Including the 3-D printed parts, the open source fracture table can be constructed for under US$3,000 in material costs, representing a 98.5% savings for commercial systems, radically increasing accessibility. The open source table can be adjusted 90–116 cm in height, tilted from +/-15 degrees, the leg height ranges from 31 to 117 cm, the arm supports and foot holder both have a 180-degree range, the foot position has a 54 cm range, and the legs can be adjusted from 55 to 120 degrees. It is mechanically adjusted so does not require electricity, however, surgical staff need to be trained on how to perform needed adjustments during surgery. The open source surgical table has verified performance for mechanical loading over 130 kg, geometric flexibility to allow for wide array of common surgeries, is radiolucent in surgical zones, and is modular and upgradeable.

## 1. Introduction

Musculoskeletal trauma is an enormous health burden worldwide. According to the World Bank, increasing access to basic surgical care would prevent 1.4 million deaths and 77.2 million disability averted life years (DALYs) per year [1]. Nine of the World Bank’s priorities of 28 essential and cost-effective surgical procedures that countries should work on making universally available are orthopedic procedures (treatment of nondisplaced fractures, closed reduction of displaced fractures, irrigation and debridement of open fractures, placement of external fixator/use of traction, escharotomy/fasciotomy, trauma-related amputations, repair of clubfoot deformity, drainage of septic arthritis, debridement of osteomyelitis) [1,2]. Surgical treatment of femur and hip fractures as well as surgery for Clubfoot repair and Dupuytren’s contractures all were found to be less costly to perform when compared to conservative treatment [3]. The Surgical Implant Generation Network (SIGN) has made low-cost implants specifically for low- and middle-income countries and trains surgeons in how to use the implants without the need for C-arm image intensification [3,4]. There are not enough surgeons trained to treat musculoskeletal issues in the developing world, and rural areas with the poorest populations are underserved and under-resourced [5]. One area where this is most clear is in the prohibitive cost of surgical equipment; specifically, surgical fracture tables, which generally cost US$200,000 have a market of US$837.1 million (2017) and is expected to rise to over US$1 billion by 2022 [6]. The used equipment donated to developing countries is often not fully functional and is not necessarily set up for current procedures and for modern surgical techniques [7]. In addition, once this donated technology fails, there is a lack of resources, both in terms of equipment (e.g. wrong voltages), supplies and personnel to fix any deficiencies and get the equipment working again [7,8].

A relatively new approach to solving these challenges is to use open source hardware licensed designs to fabricate digitally-distributed manufactured medical hardware [9–12]. 3-D printing in particular is an effective way to enable distributed manufacturing in the developing world or during humanitarian crises like a pandemic [13–16]. Such distributed 3-D printing has already been used for surgical tools, which come off of the print bed sterile [17], can be sterilized chemically, depending on the chemical compatibility of the polymer used [18], and can even be heat-sterilized with high-temperature engineering-grade thermoplastics [19]. If open source designs are freely available, surgeons and community members are free to design and modify the equipment so that it is functional for their local practice [20,21]. Previous research indicates that investment in such open hardware designs has the potential to radically reduce costs for medical facilities while offering a high return on investment for medical research funders [22]. To facilitate this means of making surgical tables more accessible, this study develops and open source surgical fracture table. The novelty of this study is to be the first attempt to provide a description of the design and manufacture of an open-source surgical fracture table that is built from materials that are widely available worldwide with 3-D printed specialty components. The bill of materials (BOM) and assembly instructions are detailed transparently. These designs are released under an open source license so that anyone may replicate, change, manufacture or use the fracture table free of intellectual property concerns. To ensure that the fracture table can perform as designed, it was tested and validated to perform mechanically to specifications. The results of these experimental validation tests are discussed in the context of reducing costs and increasing accessibility to modern medical infrastructure in the developing world.

## 2. Methods

### 2.1 Design goals

A surgical fracture table is a table that is used for applying traction to broken limbs while the body is fixed in place, allowing the surgeon to reduce the broken extremity without requiring too much assistance, and then holding the limb in this fixed and reduced position while the surgeon applies external fixation, such as a cast or splint, or internal fixation, such as a nail or plate and screws, to maintain the reduction of the extremity. Such surgical tables are expensive, but make many procedures much easier because they take on a lot of the mechanical work of the reduction and allow the surgeon finer control over the amount of traction applied and the positioning of the limb in space.

**Fig 1:**
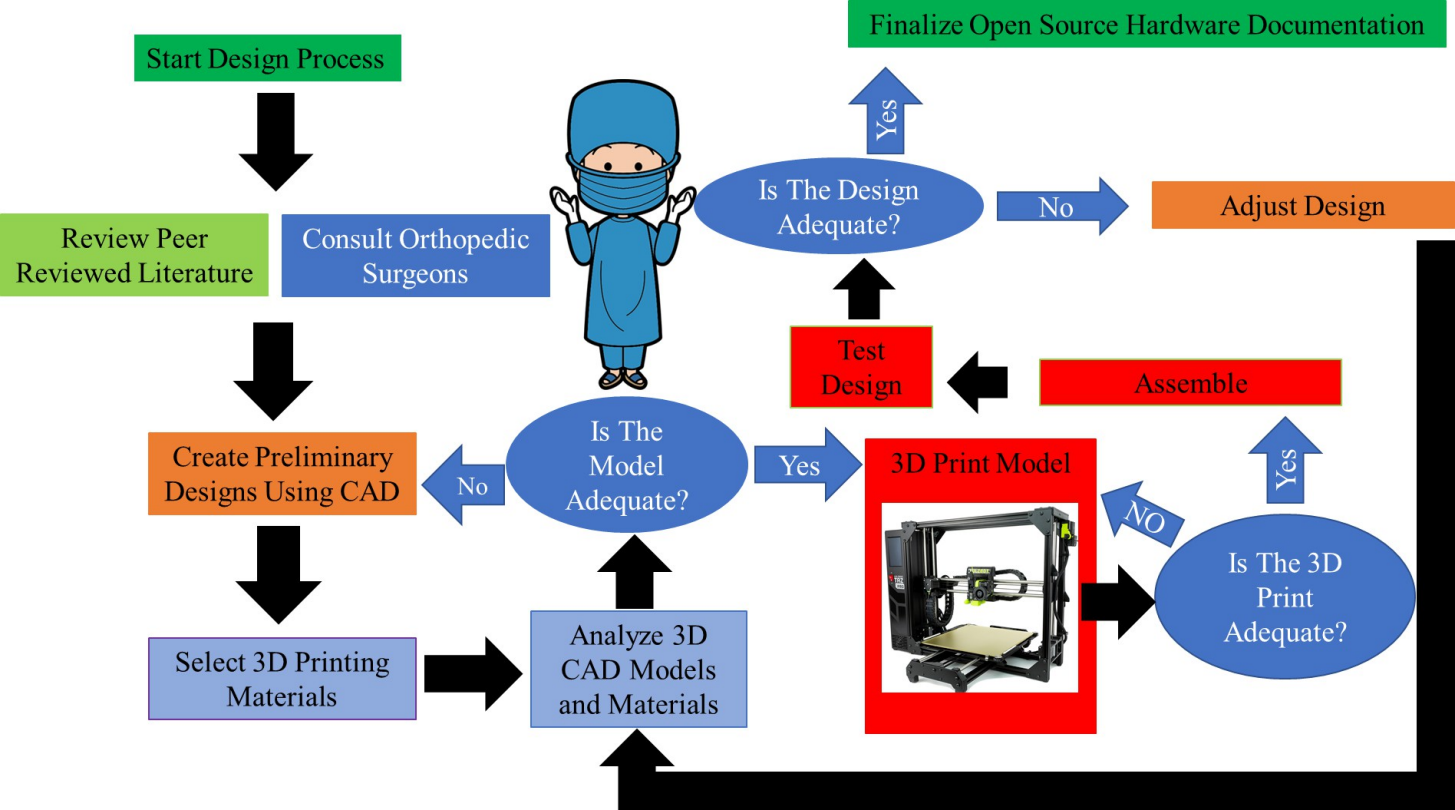
Design flow chart.

Similarly to how open source methods can be used to solve problems in medicine [23], epidemiology [24], pharmaceuticals [25] and medical information access [26], open source hardware [27,28] design methods are used here to make a digitally-replicated high-quality surgical fracture table, which i) has the ability to raise and lower to accommodate different heights of surgeon and different procedures, ii) is radiolucent to allow for C-arm image intensification or for mobile radiographic films to be taken, iii) is able to position the patient in the supine or lateral position, iv) can be operated without electricity (that is not reliably available through all of the developing world), and v) is modular to allow for traction on one or both limbs and for positioning of the injured and non-injured limbs separately. In addition, when designing a surgical table for the developing world, it is important that the materials be able to be locally-sourced and easily available, and that the quality is reproducible [29]. The table design was made to be modular to allow the ease of use of the table for multiple surgeries and for use by other surgical specialties as well, i.e. general surgery and obstetrics and gynecology. The custom parts can be manufactured on a desktop open source 3-D printer that can also fabricate its own parts [30,31]. All design files and fabrication files are released with open source licenses to enable physicians to use them [32]. The documentation is under GNU GPL 3.0 and the Hardware is licensed under CERN Open Hardware License Version 2—Strongly Reciprocal. The design flowchart for the process is shown in Fig 1.

### 2.2 Design

The design behind this table is meant to facilitate functionality and usability as well as to be easy and straight-forward to assemble. The idea behind each component is to create stability of the tabletop to prevent sway while minimizing non-fabricated parts and the use of metal (both to reduce cost but also to allow for x-ray imaging). The components that need extra durability and strength are made from high density polyethylene (HDPE) sheets [33], while the rest are 3-D printed. Smooth HDPE is chosen as the primarily building material due to its high durability, tensile strength, and chemical and moisture resistance [34]. HDPE is relatively easy to purchase and it is not relatively expensive. The 3-D printable materials used for this application are glycol modified polyethylene terephthalate (PETG) and thermoplastic polyurethane (TPU, Ninjaflex). PETG has become one of the most popular desktop 3-D printing polymers because of its relative ease to print while having high tensile strengths and impact resistance [35–37]. 3-D printed PETG was tested previously and has a mean value of the tensile modulus that ranges from 458–910 MPa [35]. Ninjaflex has been tested previously in a study of commercial FFF filaments and was found to be the most flexible commercial material, which did not break after an extension of about 800% [37]. The tensile stress for Ninjaflex at 800% extension was 12.69 MPa (average of all colors) [37]. Ninjaflex is used on the Peroneal Post Cover as it is flexible to enable comfort for the patient as well as durability [38–40].

#### 2.2.1 Bill of materials

The BOM is shown in Table 1 and all of the manufactured components are detailed in Table 2. The online repository for this project contains a visual glossary version of Table 2 to help with assembly.

**Table 1:** Surgical table bill of materials for fasteners and PVC.

Quantity	Length	Description	Cost	Source
1	15 ft.	3 inch PVC	32.60	[41]
1	8 ft.	1.25 inch PVC	5.16	[42]
68	65 mm	M12 bolt	116.97	[43]
24	50 mm	M12 bolt	63.60	[44]
12	80 mm	M12 bolt	16.32	[45]
122		M12 nut	21.75	[46]
4	thin nuts	M12 nuts	10.60	[47]
130		M12 Washer	25.96	[48]
6	1 meter	M12 x 1.75 MM threaded Rod, low strength	70.62	[49]
2	180 mm	M12 bolt	12.92	[50]
16	10.5 mm	Phillips Wood Screw 2-in	15.99	[51]

**Table 2:** Manufactured BOM.

Part #	Quantity	File Name	Material
1	1	Floor Base Support	HDPE
2	1	Back Base Panel	HDPE
3	1	Front Base Panel	HDPE
4	2	Side Base Panel	HDPE
5	4	Base 3in pipe	19.5 in x 4 PVC
6	12	3_INCH_PVC_to_HDPE_Sheets_Outside	3D printed material
7	1	Bottom of Support Structure	HDPE
8	1	Jack Top HDPE	HDPE
9	2	Jack Side Screws	HDPE
10	2	Jack Side Insert	HDPE
11	1	Top of Support Structure	HDPE
12	6	Lower_Arc_for_Table_Bracket	3D printed material
13	2	Lower_Arc_for_Table_Angle	3D Printable Material
14	2	Upper_Arc_for_Table	3D Printable Material
15	2	Table Top Support	HDPE
16	6	Table Top Bracket	3D Printable Material
17	1	Table Top	HDPE
18	6	arm-rest_mounting_pin	3D Printable Material
19	2	Arm Attachment	HDPE
20	2	Arm Rest	HDPE
21	6	arm-rest_mounting_pin_clip	3D Printable Material
22	1	Distancing Brace	HDPE
23	2	Leg Pivot X-Axis	3D Printable Material
24	2	Leg Multi Pivot	3D Printable Material
25	2	Leg Pivot to Pipe	3D Printable Material
26	2	Table Support Pin	3D Printable Material
27	2	Pelvic Post Mount Pin	3D Printable Material
28	1	Peroneal Support	HDPE
29	1	Peroneal Brace	HDPE
30	1	pelvic_post_mount	3D Printable Material
31	1	Peroneal Post Cover	NinjaFlex
32	1	Pelvic_post_mount_pin_NEW	3D Printable Material
33	2	1.25in pipe	72 in x 2 PVC
34	2	Leg Vertical Positioner	3D Printable Material
35	2	3in PVC	48 in x 2 PVC
36	4	Vertical Pvc Leg Support NEW	3D Printable Material
37	2	Foot Tensioner	3D Printable Material
38	2	Lead Screw For Foot Pedastal	Metal
39	2	leadscrew_handle	3D Printable Material
40	2	foot-rest_mount_with_angle_fix	3D Printable Material
41	2	Foot-Rest	3D Printable Material
42	2	Friction End	3D Printable Material
43	1	Peroneal Post Cover Base	NinjaFlex
44	As needed	Person to Table Holder Tall	3D printed material
45	As needed	Person to Table Holder Short	3D printed material
46	As needed	Person to Table Holder Tall Half	3D printed material
47	As needed	Person to Table Holder Tall Quarter	3D printed material
48	As needed	Person to Table Holder Short Half	3D printed material
49	As needed	Person to Table Holder Short Quarter	3D printed material
50	1	Leg Top Holder	3D printed material
51	1	Leg Top HDPE	HDPE
52	2	Foot Fastener	3D printed material
53	2	Jack to Table Support	HDPE

These components were printed on a RepRap-class fused filament fabrication-based 3-D open source 3-D printer (Taz 6, Fargo Additive Manufacturing Equipment 3D). They used open source Lulzbot Cura v.2.6.23 default settings for PETG and ninjaflex (e.g. 60mm/s for PETG and 15mm/s for ninjaflex). The default profile for PETG is available [32]. The infill parameters used for printing PETG are detailed in Table 3, based on the mechanical loads the parts would experience in normal use. Parts were printed on a standard 0.5 mm nozzle, but can also be more rapidly produced with a 1.2 mm MOAR Struder. It should be noted; however, these printed parts could be manufactured on any RepRap-class 3-D printer that has the build volume to make the parts. Parts that required more structural integrity used a higher percent fill and all parts were at least 50% fill as can be seen in Table 3. The overall cost of the materials for the open source fracture table is shown in Table 4.

**Table 3:** 3-D printed components.

Description	Quantity	Infill (%)	Grams of PETG	Print Time (hrs) Regular Nozzle 0.38 layer height	Total PETG	Total Print Time (hrs)	Print Time (hrs) MOAR struder head 0.6 mm layer height	Moar Struder Total Print Time (hrs)
3 INCH PVC to HDPE Sheets Outside	12	75	147	5.75	1764	69.00	2.32	27.80
Lower Arc For Table Bracket	6	100	83	3.23	498	19.40	1.33	8.00
Lower Arc for Table Angle	2	75	1338	41.73	2676	83.47	16.88	33.77
Upper Arc for Table	2	75	295	10.12	590	20.23	4.95	9.90
Table Top Bracket	6	75	147	5.25	882	31.50	2.12	12.70
Arm Rest Mounting Pin	6	60	34	1.58	204	9.48	0.30	1.80
Arm Rest Mounting Pin Clip	6	60	16	1.12	96	6.72	0.18	1.08
Leg Pivot X axis Updated	2	50	213	7.65	426	15.30	3.60	7.20
Leg Multi Pivot	2	60	547	17.47	1094	34.93	7.30	14.60
Leg Pivot to Pipe	2	60	281	9.78	562	19.57	3.88	7.77
Table Support Pin	2	50	53	3.63	106	7.26	0.70	1.40
Peroneal Post Mount Pins	8	100	53	2.40	424	19.20	0.68	5.47
Peroneal Post Center Pin	1	100	156	6.77	156	6.77	1.92	1.92
Peroneal Post Cover	1	50	535	16.32	535	7.65	6.65	6.65
Pelvic Post Mount Pin	2	80	15	1.15	30	2.30	0.27	0.54
Leg Vertical Positioner	2	100	470	21.67	940	43.33	6.17	12.33
Vertical PVC Leg Supports	2	100	203	9.38	406	18.76	3.15	6.30
Foot Tensioner	2	60	326	13.35	652	26.70	5.38	10.77
Leadscrew Handle	2	70	20	1.68	40	3.36	0.32	0.64
Foot Rest Mount with Angle Fix	2	50	178	12.55	356	25.10	2.35	4.70
Foot Rest	2	50	164	11.67	328	23.34	2.30	4.60
Friction End	2	50	9	0.72	18	1.44	0.15	0.30
PVC Pin Clips	4	100	2	0.18	8	0.73	0.03	0.13
PVC Pins	4	100	15	0.77	60	3.07	0.22	0.87
Clamp for Jacks	4	100	177	7.93	708	31.73	3.00	120.00
				**Totals**	**13559**	**530.34**		**182.31**
					**grams**	**hours**		**hours**

**Table 4:** Overall costs (2022 values).

Component	Cost in USD	Sources
Hardware(fasteners) + PVC	$392.49	[41–51]
HDPE Plastic	$2173.39	[52]
PETG for 3DP	$307.86	[53]
Jacks	$64.99	[54]
Total	**$2,938.73**	

All 3-D printable designs are made available on the Open Science Framework in IPT CAD format and STEP files for editing and STL files for direct 3-D printing. A pad, not shown in the CAD is meant to be placed on top of the table, chosen by the owner to give the patient the best comfort during surgeries.

#### 2.2.2 Assembly

Abbreviated assembly instructions follow. Step by step assembly instructions are available on the Open Science Framework repository for this article [32].

**Fig 2:**
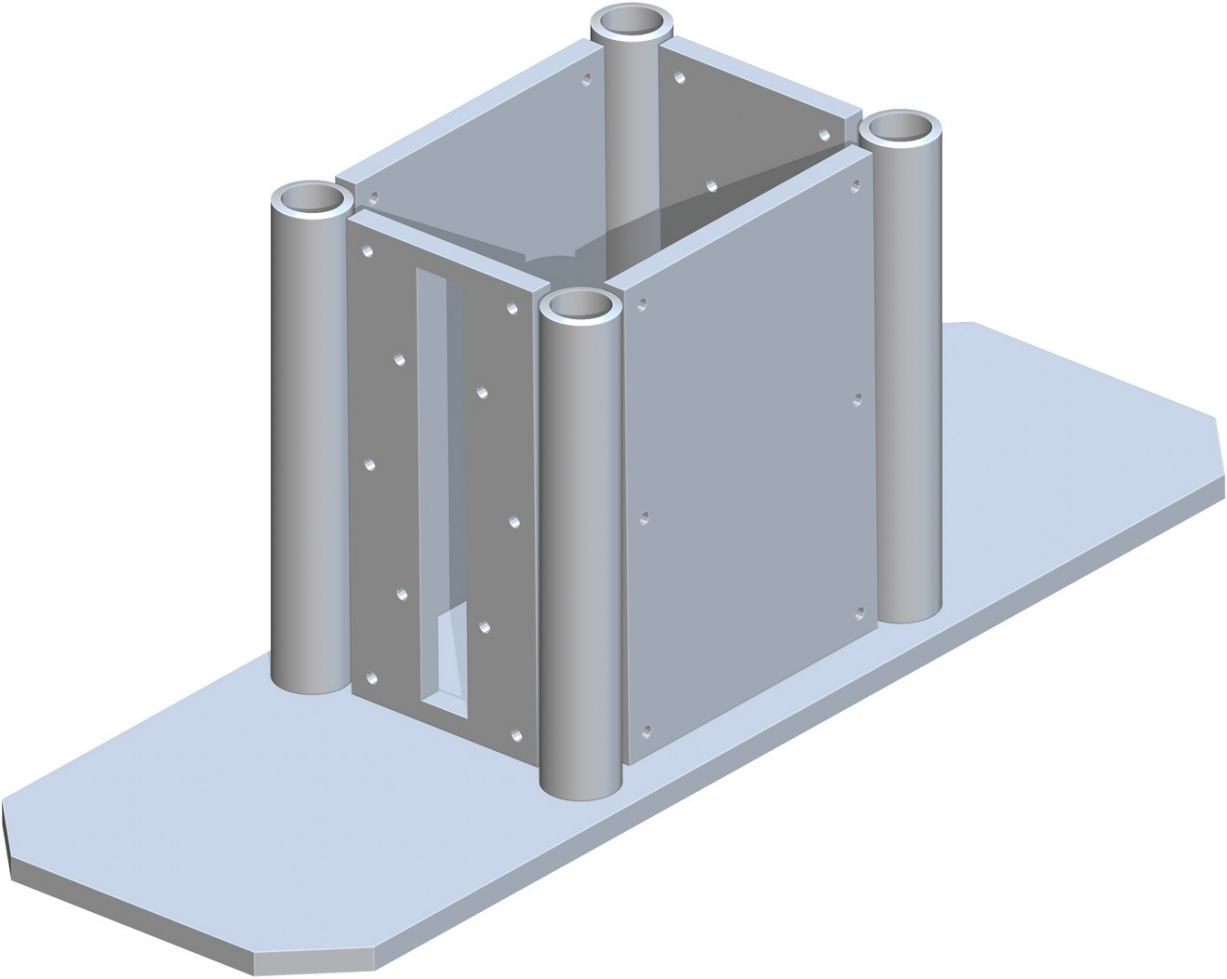
Adding four Base 3in Pipes to the corners of the Base Panels.

*Base support structure assembly*. The base support structure assembly begins with part 1, the Floor Base Support. This can be placed anywhere but must be grounded, as it is the key component that enables the table from being too wobbly or moving too much. Next, the Back Base (part 2) and Front Base (part 3) Panels are placed on top part 1. Part 2 is placed 18 inches flush from the back side of part 1 and 4.5 inches flush from the side. Part 3 is placed 17.583 inches flush from the front side of part 1 and 4.5 inches flush from the side. Part 2 and 3 should be parallel with each other. The Side Base Panels (part 4) are then placed, one at a time. The length of part 4 is 2 inches flush from the side of part 1. The depth of part 4 is 19.833 inches flush from the front side of part 1. A Base 3in Pipe (part 5) is placed at each corner intersection of the Base Panels. Part 5 is 0.5 inches tangent to each panel in the corner. This representation is shown in Fig 2.

**Fig 3:**
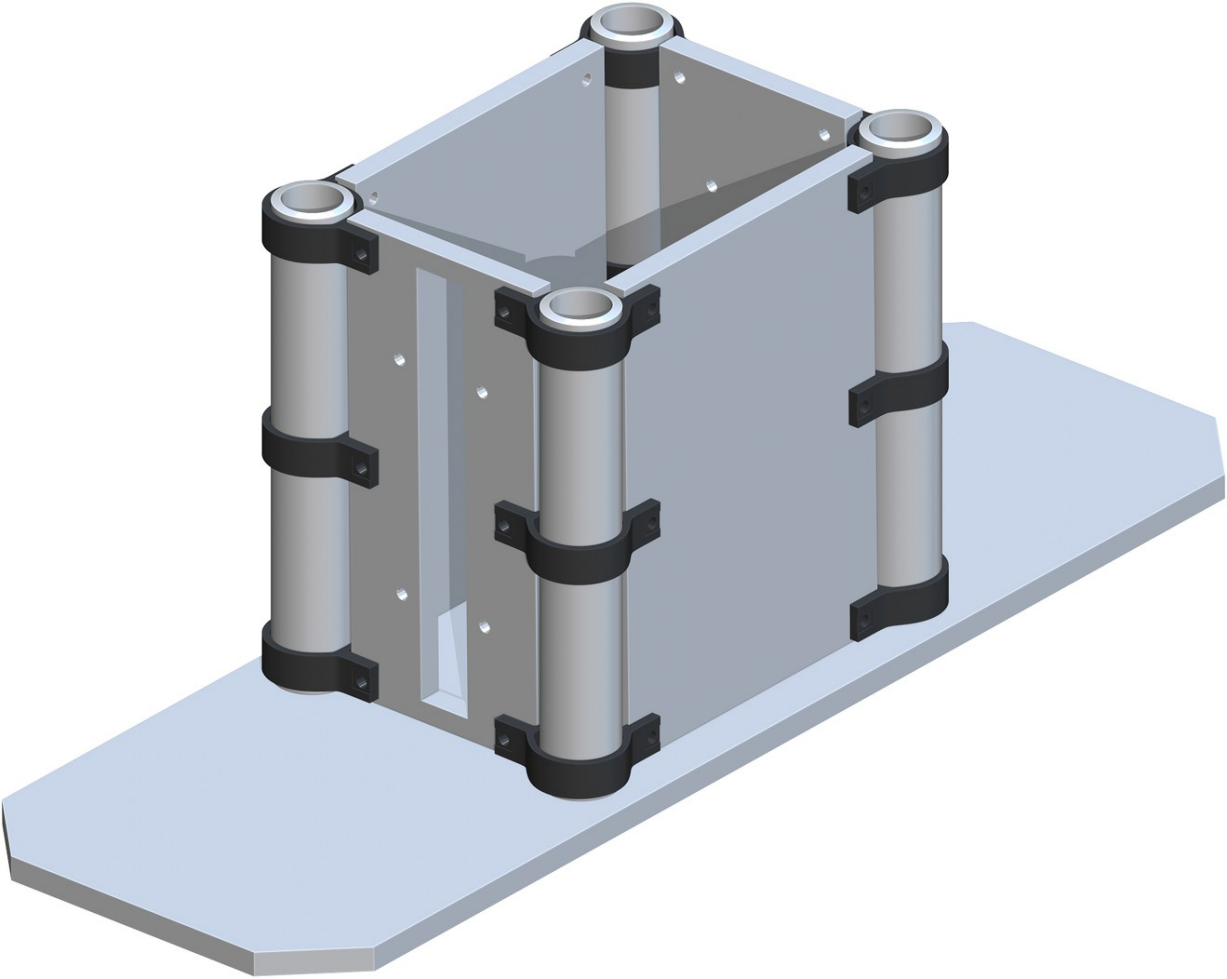
Total 3_INCH_PVC_to_HDPE_Sheets_Outside on each Base 3in Pipe.

The 3_INCH_PVC_to_HDPE_Sheets_Outside (part 6) are then placed around part 5. The bottom one will have each of the drill holes aligned with the drill holes in the Base Panels. Three will be placed on each Base 3in Pipe. There will be a total of 12 of part 6’s, which is found in Fig 3.

**Fig 4:**
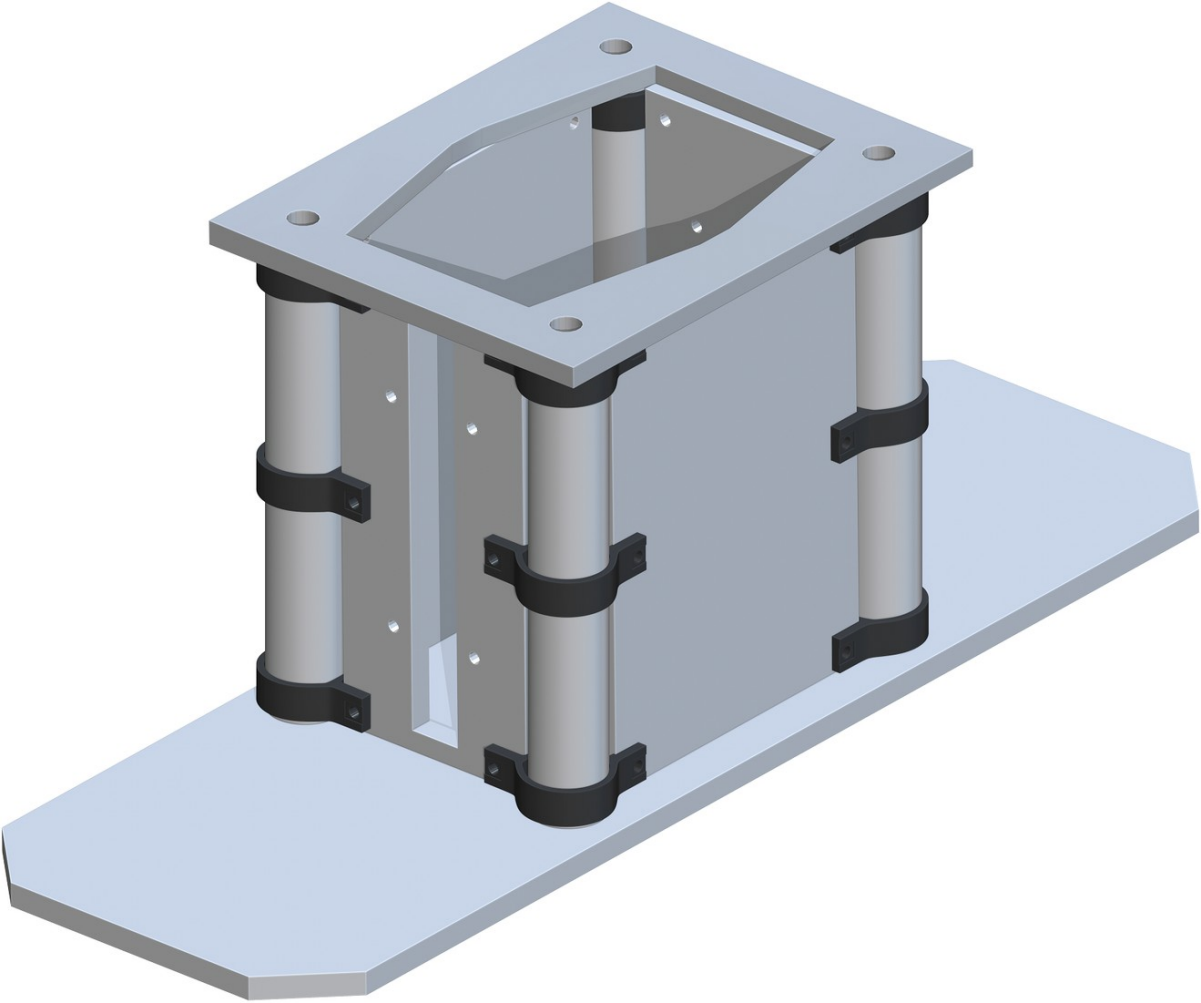
Addition of bottom of support structure to the base.

*Jack support assembly*. The Bottom of Support Structure (part 7) is added flush to the tops of each Base Panel and centered. The holes in each corner directly center align with part 5. This is shown in Fig 4.

**Fig 5:**
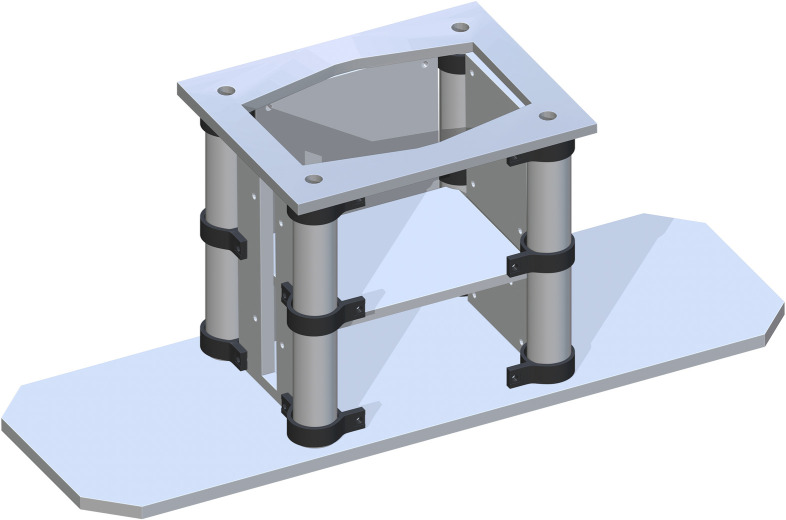
Addition of Jack top HDPE to center of base assembly.

A Jack Top HDPE (part 8) is added mate to the top of part 1 six inches above. The ends are then mate to parts 2–4. This will sit on top of the Jack in the center of the base. This is shown in Fig 5.

**Fig 6:**
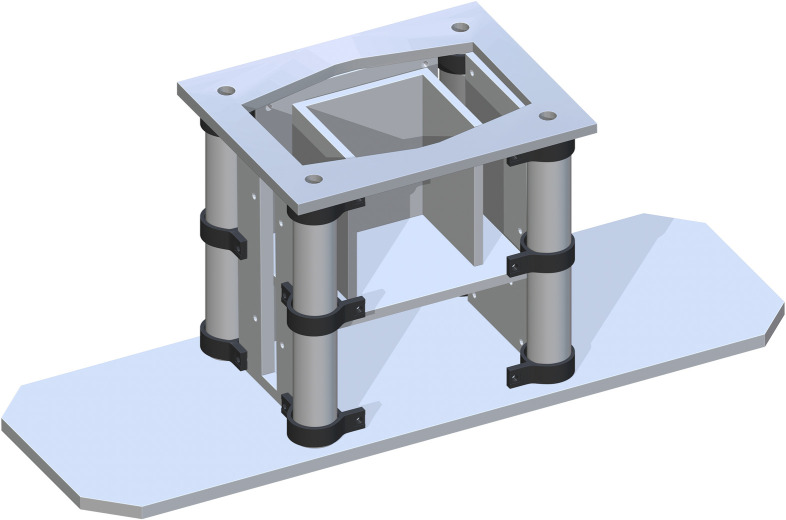
Addition of one side Jack insert.

The Jack Side Screws (part 9) are then added mate with part 8 on top. It is flush 2.125 inches from part 2 inward. Another panel is added opposite with the same flush to part 3. The Jack Side Insert (part 10) is the next to be added. They are mate to the insides of part 9 and center aligned with the screw holes. The side is flush to the size of part 8. Two of part 10 are added to each side of part 8. The addition of one is shown in Fig 6.

**Fig 7:**
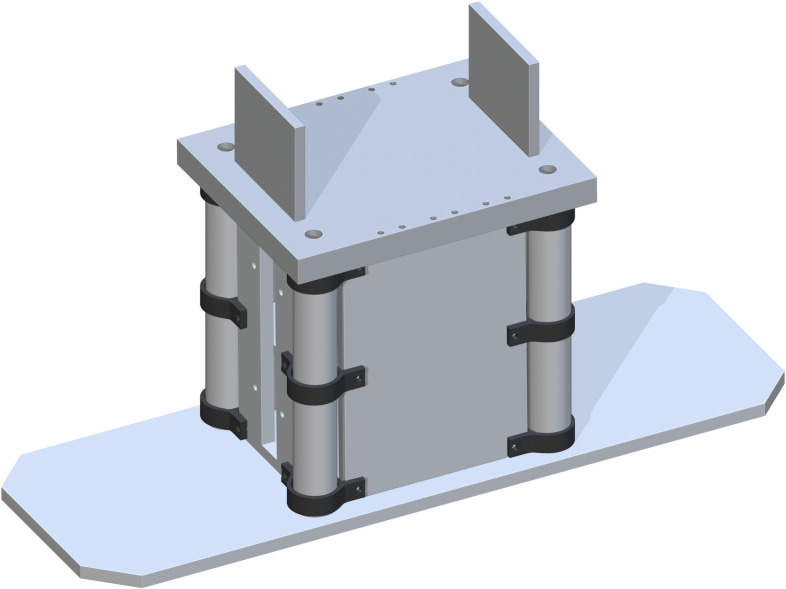
Completing the base support structure by adding the Top Support Structure.

A Top Support Structure (part 11) is then added flush to the tops of each Base Panel and centered. Each drill hole in the corners of part 11 is to be center aligned with each center of part 5. This ensures the ability for best attachment and stability of the base support structure, with the addition of the Jack to Table Support (part 53), centered in the cut rectangles of part 11. This is shown in Fig 7.

**Fig 8:**
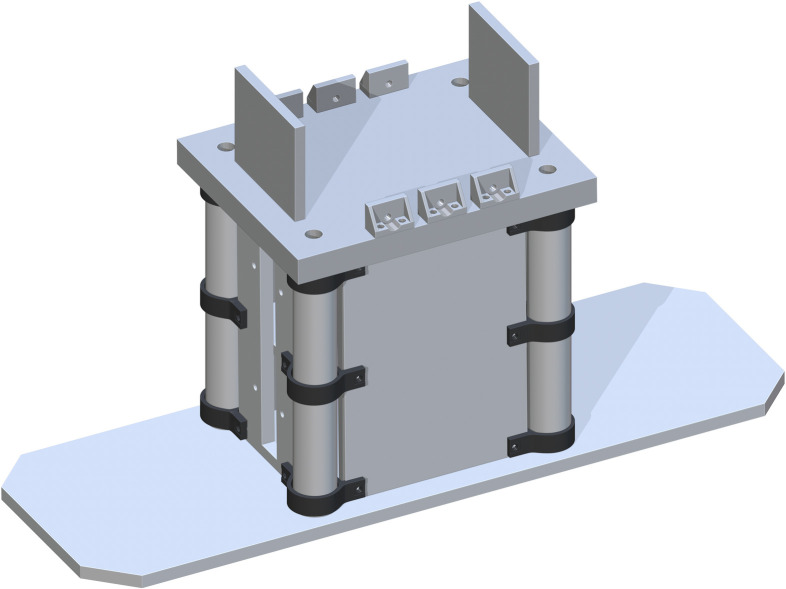
Finishing the addition of Lower_Arc_for_Table_Bracket’s.

*Table top assembly*. The Lower_Arc_for_Table_Bracket (part 12) is attached to part 11 in one dual set of drill holes. The front side of part 12 should be flush with the length side of part 11. Both holes should be aligned in their centers. Repeat this process for six brackets along the edges of part 11, shown in Fig 8.

**Fig 9:**
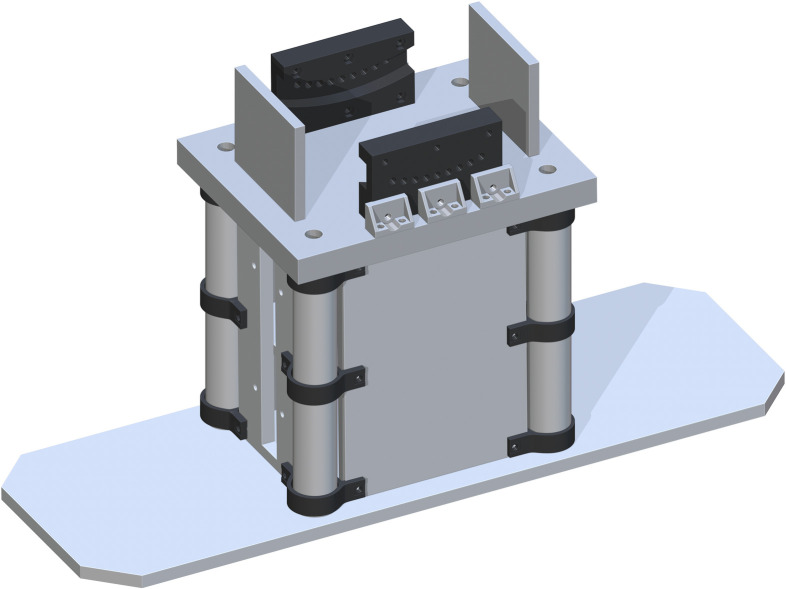
Attaching two Lower_Arc_for_Table_Angle to Lower_Arc_for_Table_Bracket.

The Lower_Arc_for_Table_Angle (part 13) is attached mate to the backside of three of the brackets on one side of part 11. The center drill holes in part 12 should be aligned with the center holes of part 13. Repeat this process on the other side of the table with the other three brackets. This is shown in Fig 9.

**Fig 10:**
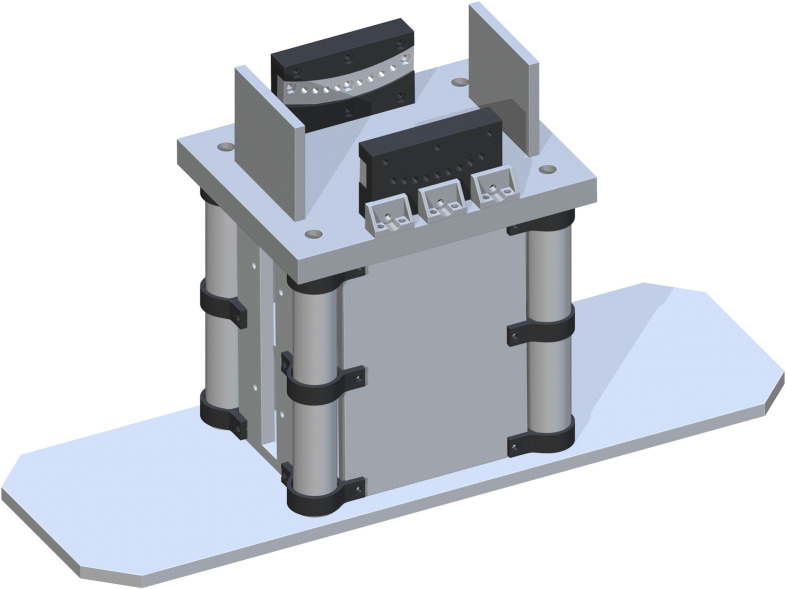
Adding two Upper_Arc_for_Table to both Lower_Arc_for_Table_Angle.

The Upper_Arc_for_Table (part 14) is then added inside of part 13. The center drill holes should align within the arc of holes of part 13. Each side of part 14 should be flush with the sides of part 13. Add these to each of part 13, shown as the grey (part 14) inside the black (part 13) in Fig 10.

**Fig 11:**
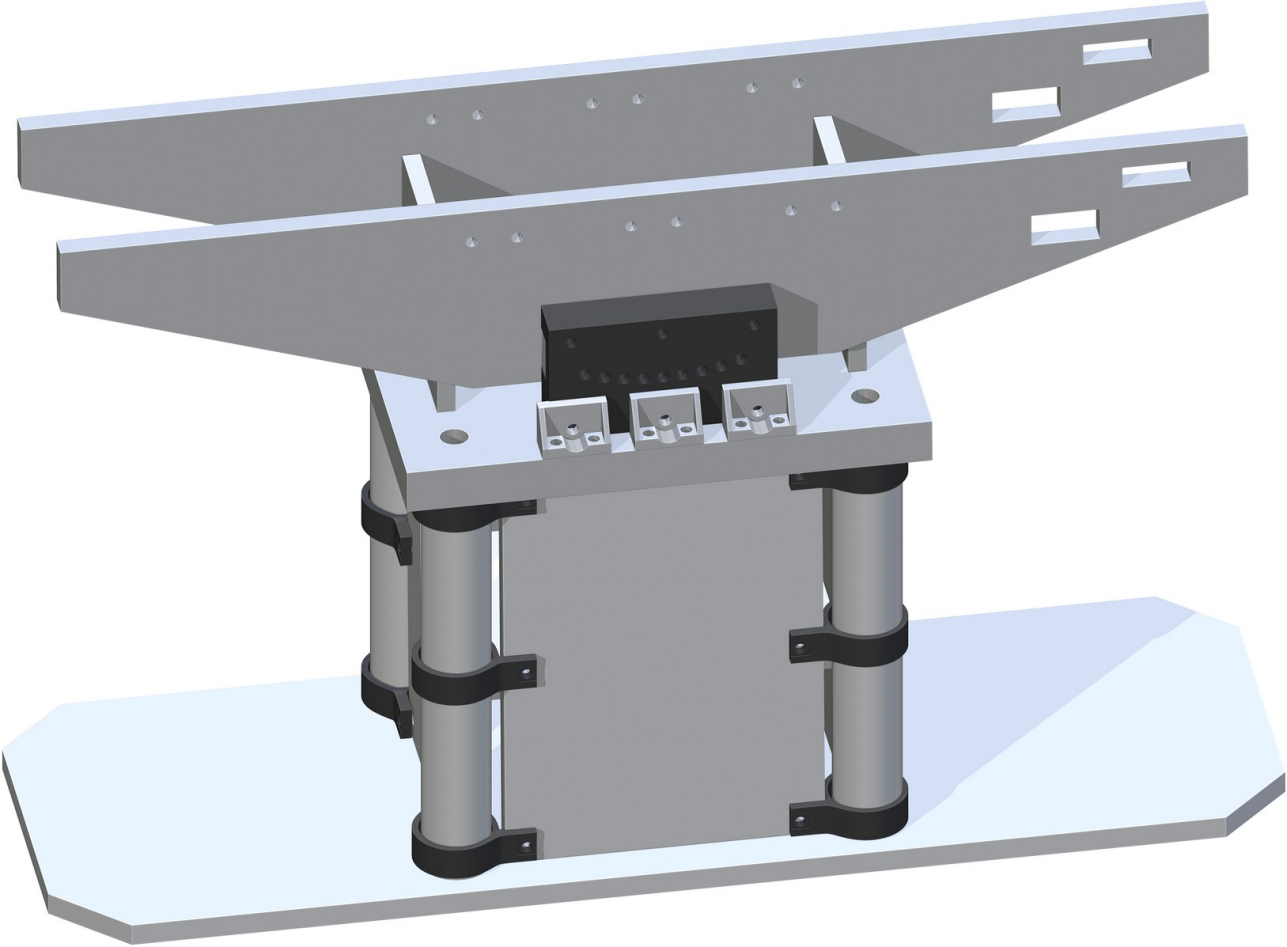
Final view of both Table Top Support components.

The Table Top Support (part 15) is attached to part 14 and center aligned with the three largest drill holes. The side of part 15 is 21 inches flush with the side of part 13 to ensure equal distribution. This process is repeated with the other side, shown in Fig 11

**Fig 12:**
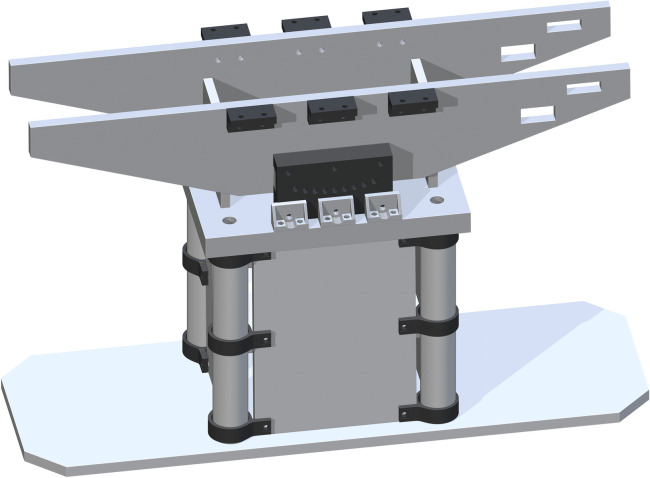
Final attachments of table top bracket.

Then the Table Top Bracket (part 16) is attached to the double drill hole at the top of the Table Top Support. The holes on part 16 are center aligned with the holes on part 15. Three brackets will attach to the outside of each part 15. The top of part 16 will be flush with the top of part 15. Three more of part 16 will attach to the opposite side of the other part 15, shown in Fig 12.

**Fig 13:**
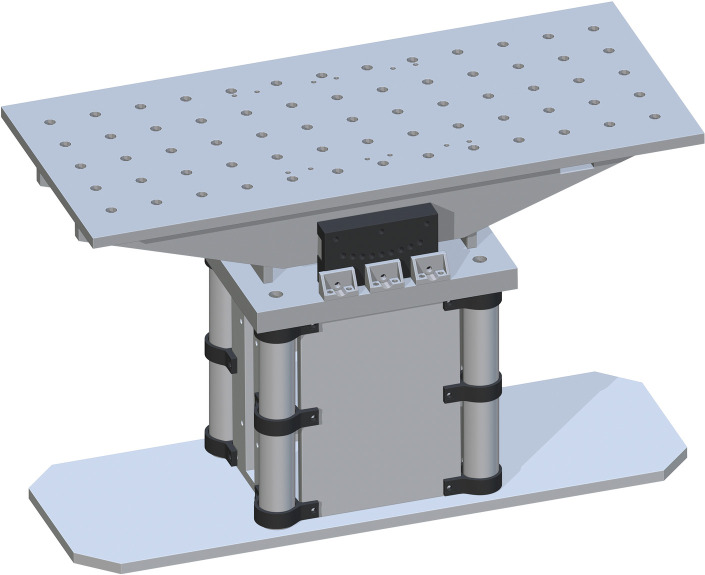
Completing the Table Top assembly by adding the Table Top.

Lastly, the Table Top (part 17) is placed on top. The drill holes on part 17 are to center align with the top drill holes of part 16. This will fit on top perfectly. This final assembly is shown in Fig 13.

**Fig 14:**
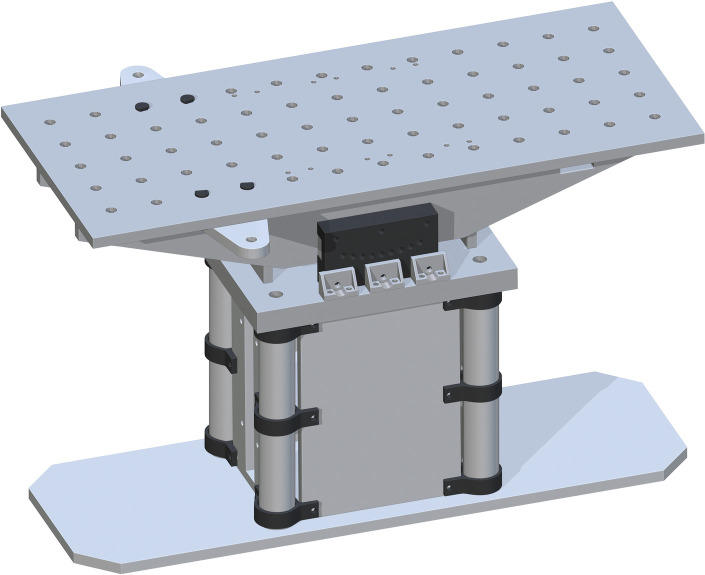
Addition of Arm Attachments to Table Top.

*Arm assembly*. The arm-rest_mounting_pin (part 18) is the next to be added on top of part 17. These pins can be added in any 2 consecutive outside drill holes. Part 18 will be center aligned. The two pairs or four pins are directly opposite one another. The Arm Attachments (part 19) are next to be added to part 17. The tops of part 19 are mate to the bottom of part 17. The two drill holes close together will be center aligned with the two of part 18. This is repeated on both sides of part 17, shown in Fig 14.

**Fig 15:**
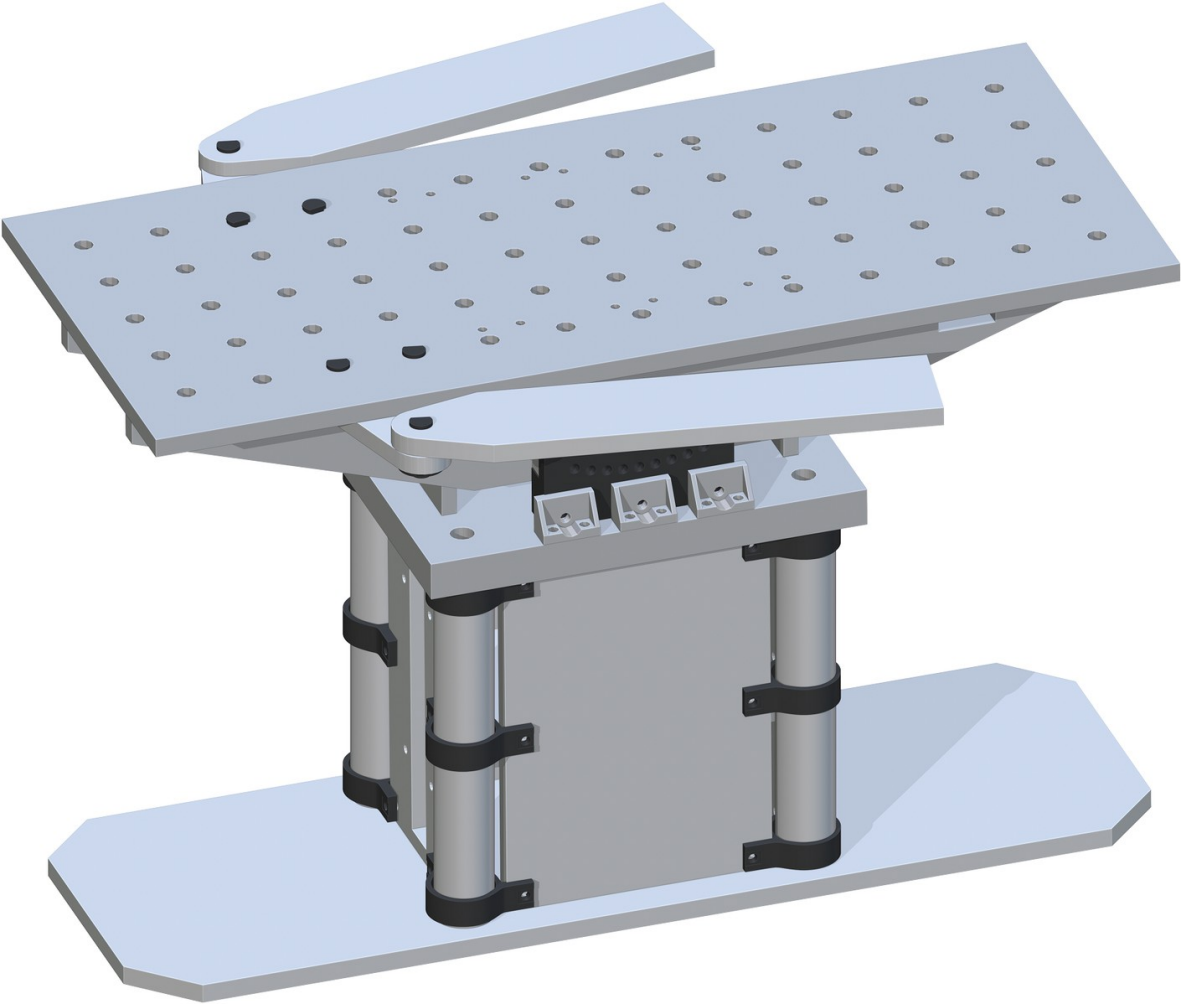
Final two arm-rest_mounting_pin holding Arm Rests in place.

The Arm Rest (part 20) is now added to the Arm Attachments. The bottom of part 20 is mate with the tops of part 19. The holes at the top of part 20 are center aligned with the single hole of part 19. Both Arm Rests are attached, shown in Fig 15. Two more of part 18 are added into those holes to keep part 20 from dislocating to part 19.

The arm-rest_mounting_pin_clip (part 21) is added as a washer to hold part 18 from easy removal. Part 21 is mate to the bottom of all components in the arm-rest_mounting_pin slit.

**Fig 16:**
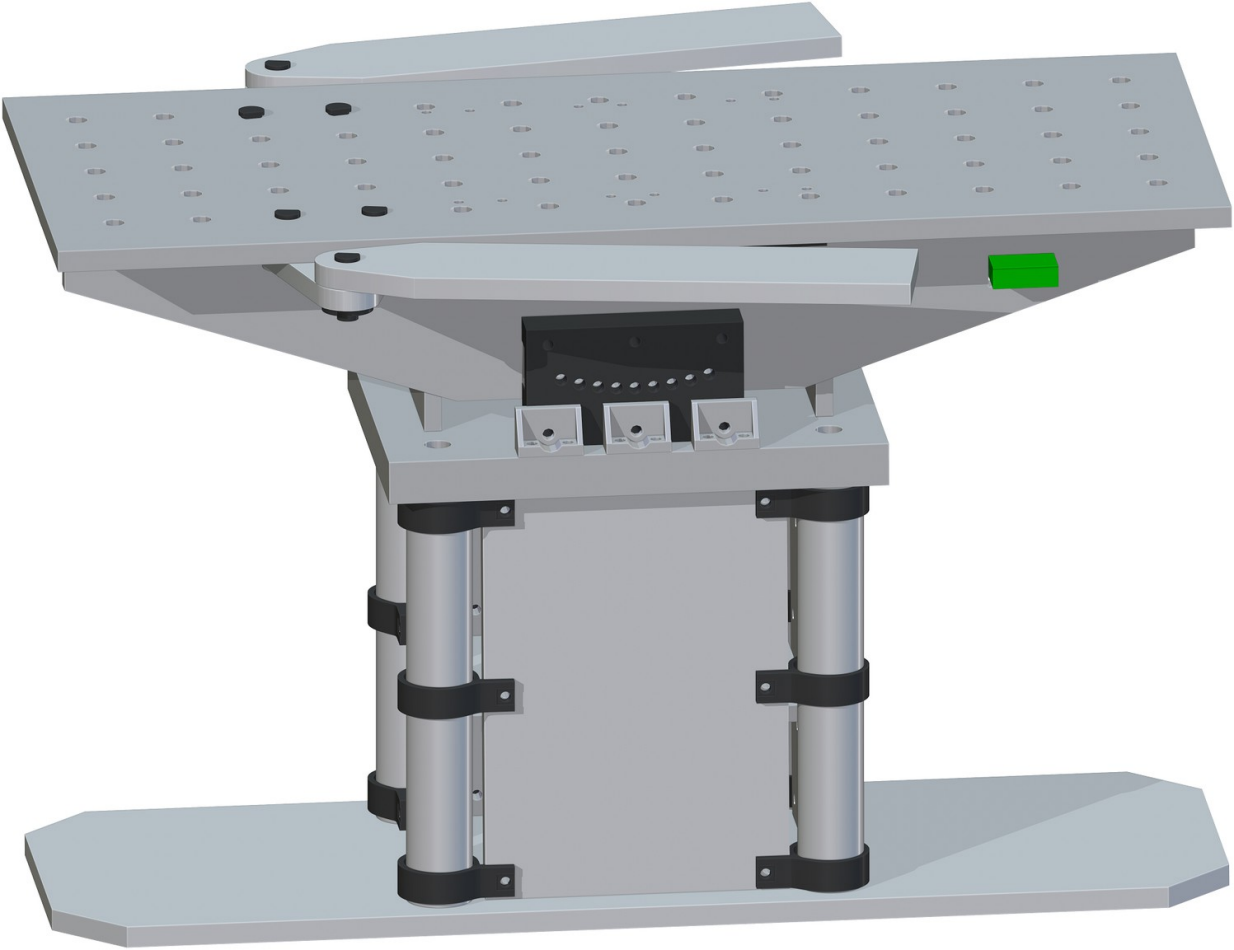
Addition of Distancing Brace through the Table Top Support.

*Leg attachment assembly*. The Distancing Brace (part 22) is inserted through the lower rectangular openings on part 15. The top of part 22 is mate with the top of the opening of part 15. This is shown as the green component in Fig 16.

**Fig 17:**
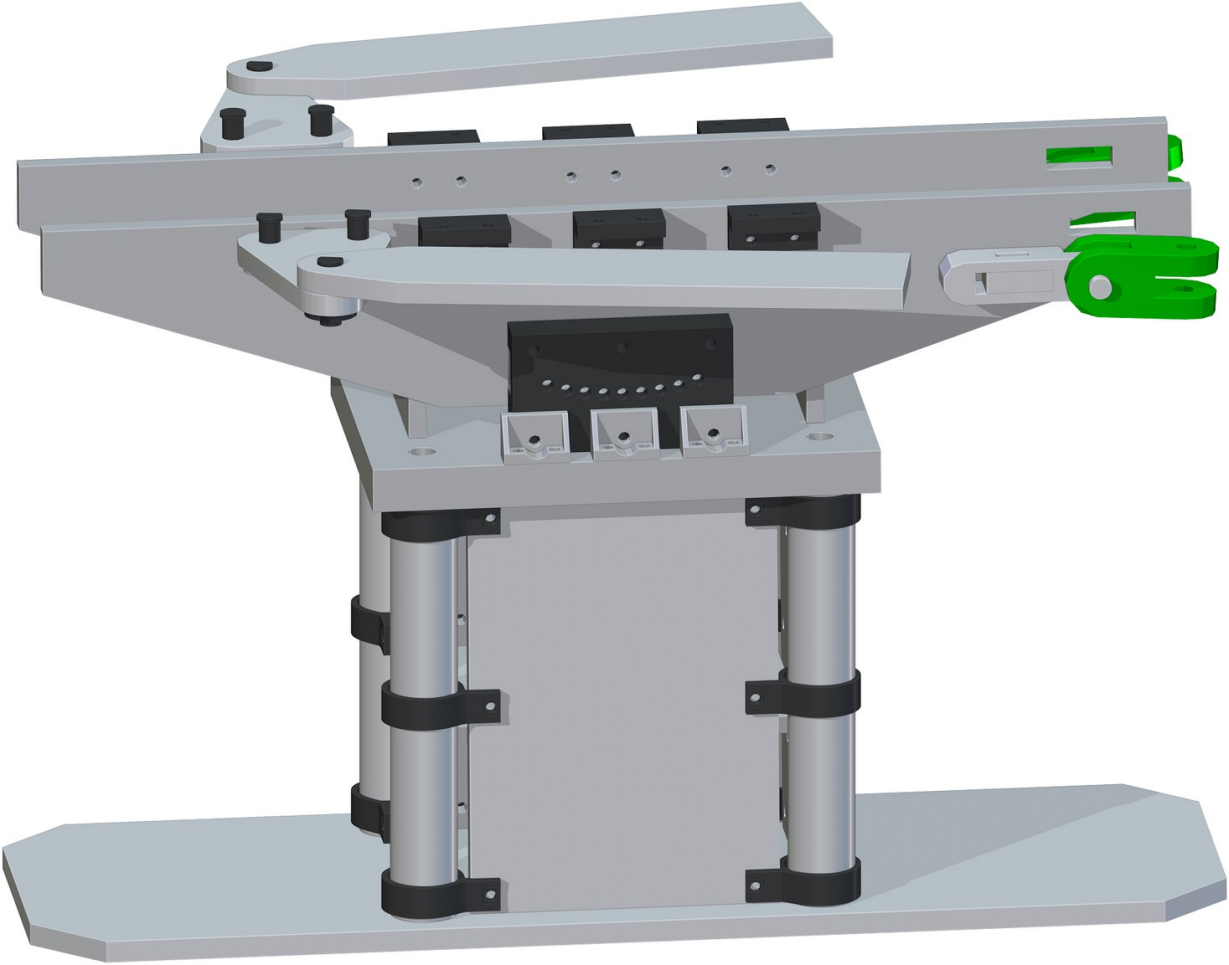
Addition of Leg Multi Pivot to Leg Pivot X-Axis.

The Leg Pivot X-Axis (part 23) has a rectangular opening, where the end of the part 22 will be flush with the outside of part 23. They will be attached to both sides of part 22, with the whole facing toward the leg side of the table. The Leg Multi Pivot (part 24) is then attached to the Leg Pivot X-Axis. This component is designed to be placed on either way, shown as the green component in Fig 17. The hole of part 24 and of part 23 will be center aligned. Two of part 24 will be attached to each part 23.

Then, the Leg Pivot to Pipe (part 25) is attached to the Leg Multi Pivot (part 24). The circular hole in part 25 is to be center aligned with the open hole in part 24. Part 25 has the ability to turn back and forth.

**Fig 18:**
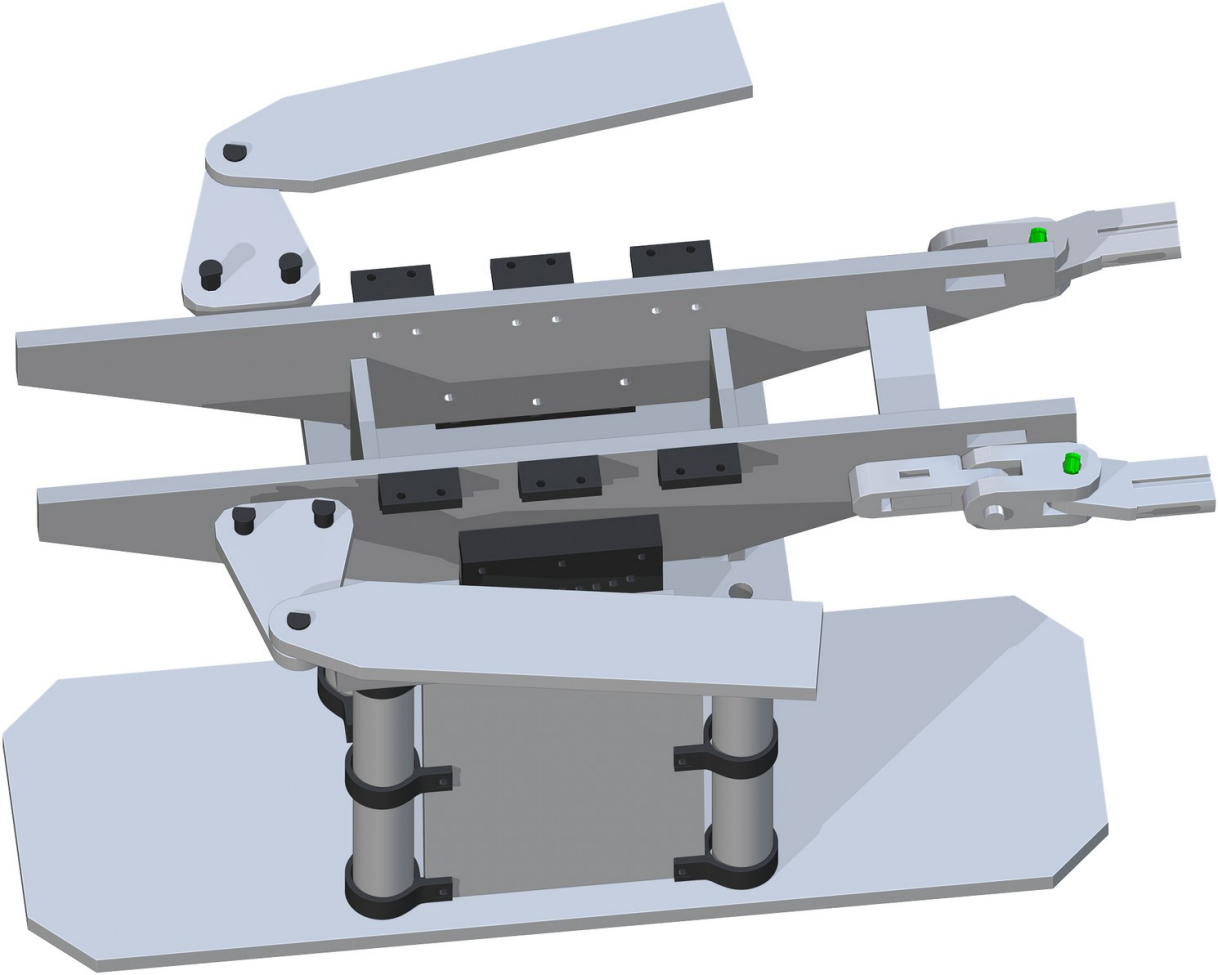
Addition of final component Pelvic Post Mount Pin into Leg Attachment Assembly.

The Table Support Pins (part 26) were then added at the horizontal holes through parts 23 & 24. This action is repeated on the opposite side of the table. The last addition to the Leg Attachment Assembly is the Pelvic Post Mount Pin (part 27). This is center aligned through parts 24 & 25, as shown as the green component in Fig 18.

**Fig 19:**
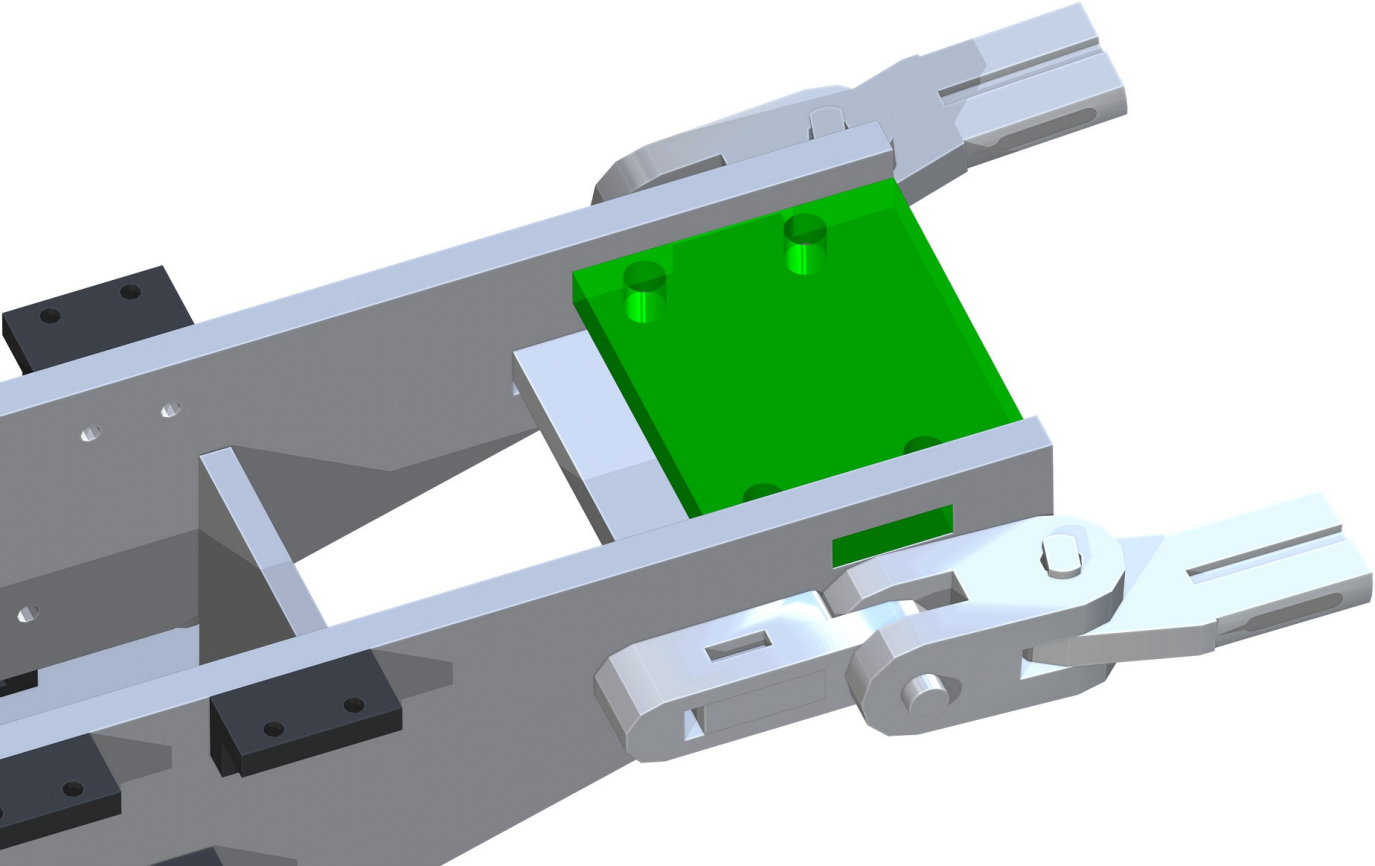
Addition of Peroneal Support to the Table Top Support.

*Peroneal support assembly*. The Peroneal Support (part 28) is then added in the open rectangle of the Table Top Support. The hole of part 28 that overlaps with the rectangular extension is closest to the leg end of the table. The addition of this part is shown as the green component in Fig 19.

**Fig 20:**
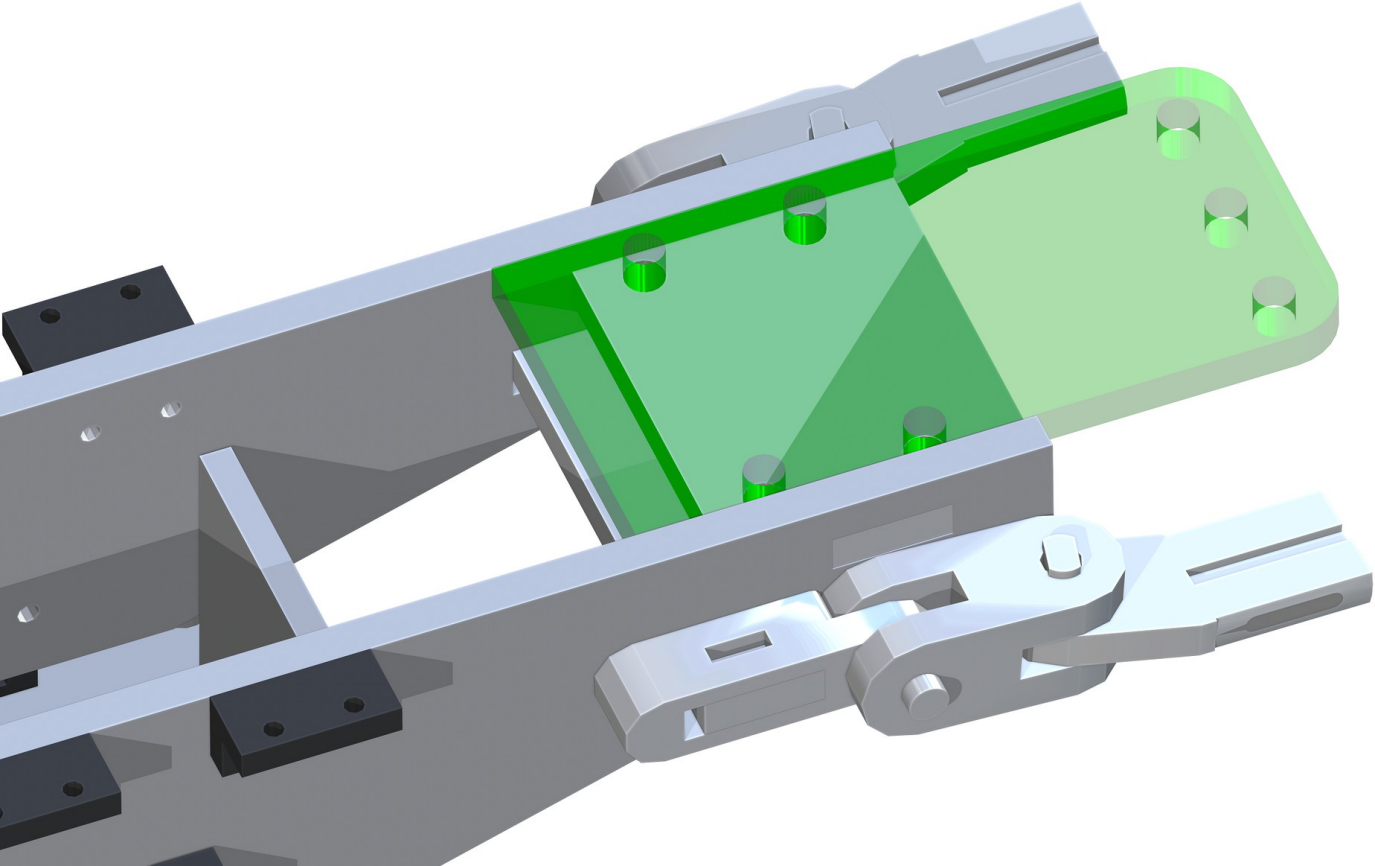
Addition of Peroneal Brace to the Peroneal Support.

The Peroneal Brace (part 29) is added on top of part 28. The dual holes are center aligned with part 28 and part 29 can be adjusted with two different distances. This is shown as the green component in Fig 20.

**Fig 21:**
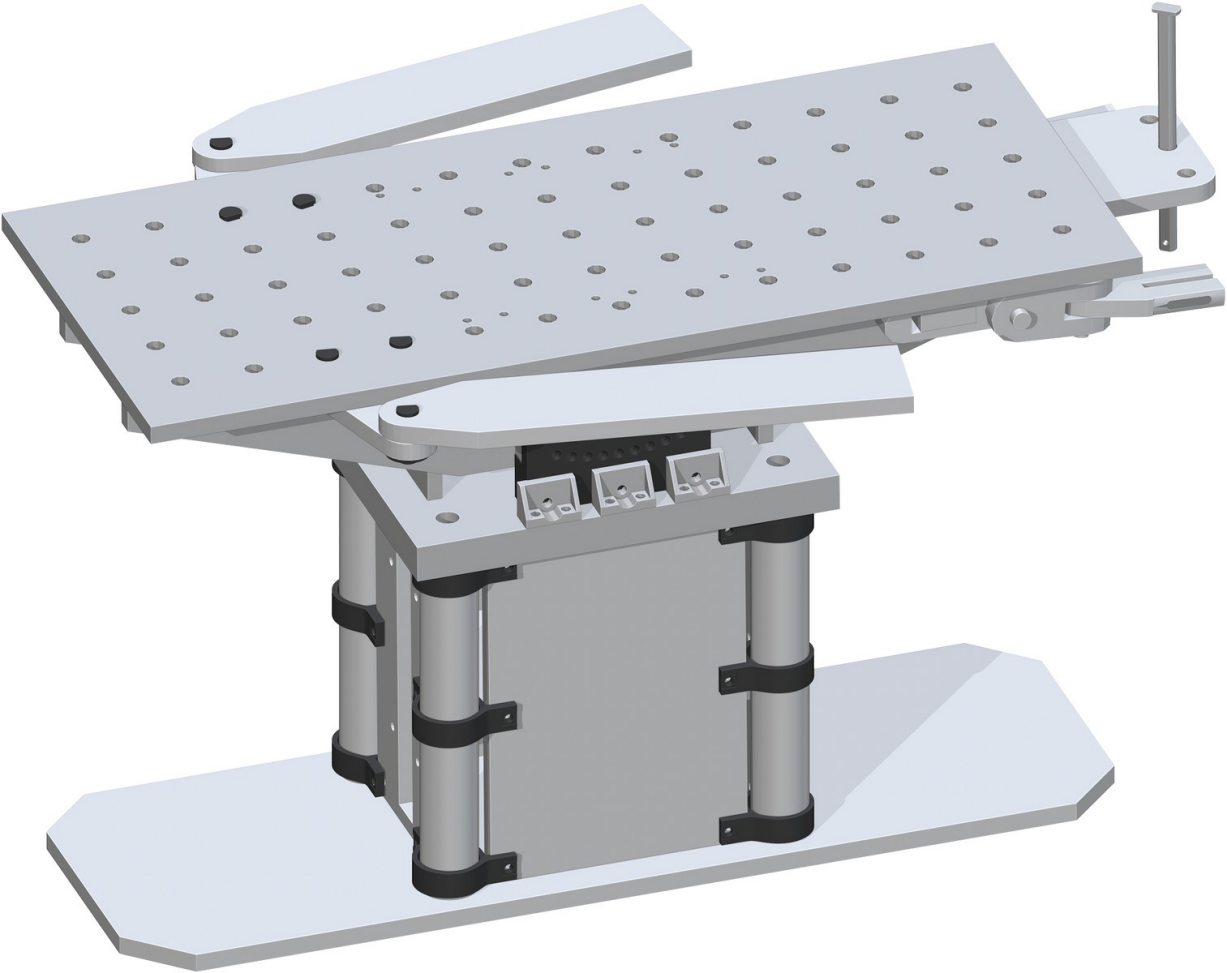
Addition of pelvic_post_mount to Peroneal Brace.

The pelvic_post_mount (part 30) is a long thin rod that is inserted into any of the three holes on the end of part 29. It is center aligned with each individual hole, shown in Fig 21.

**Fig 22:**
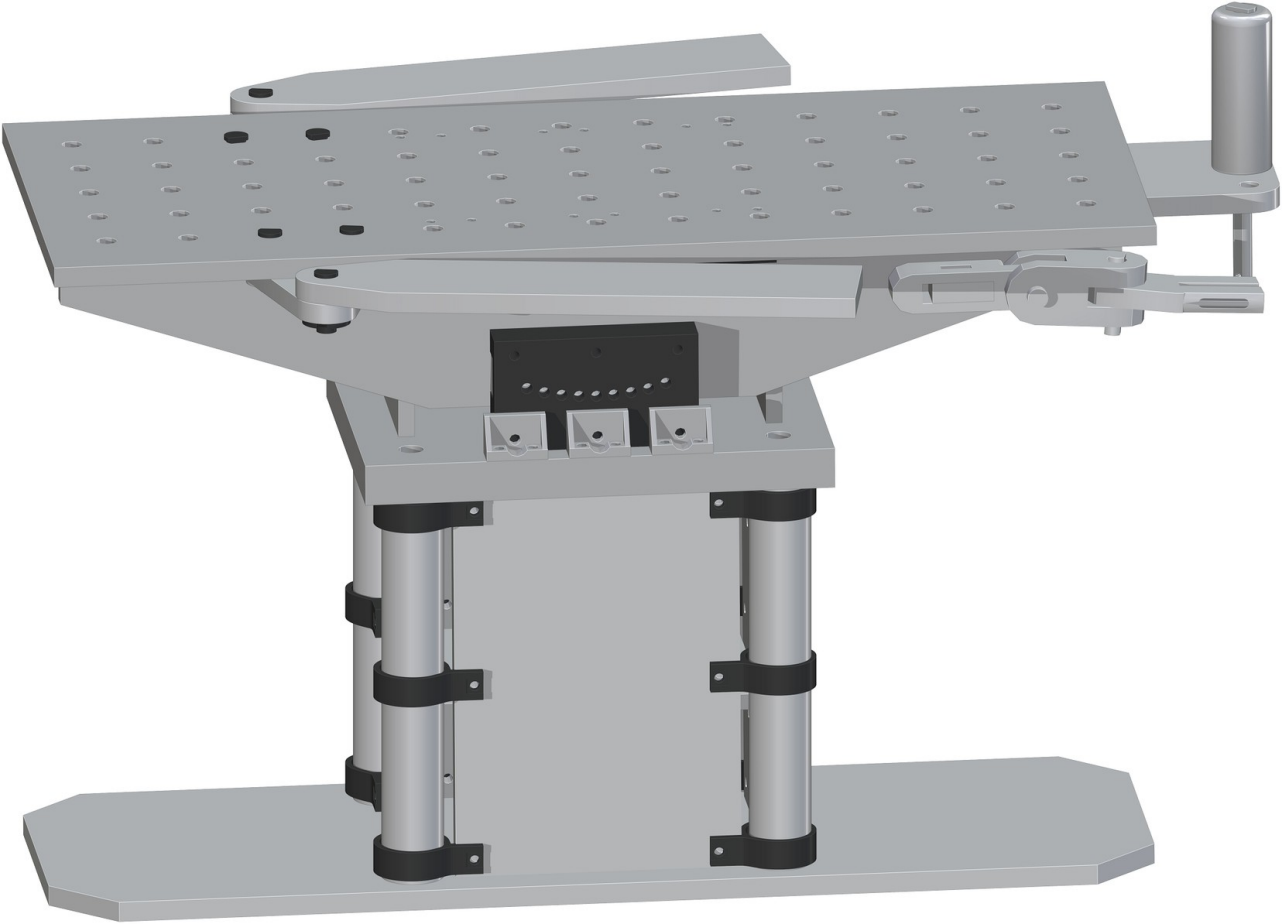
Addition of Peroneal Post Cover around the pelvic_post_mount.

The Peroneal Post Cover (part 31) is center aligned with part 30. Part 43, the Peroneal Post Cover Base is an additional print to allow part 31 an additional 6 inches in height. This is shown in Fig 22. The base of part 31 is mate with part 29.

The pelvic_post_mount_pin (part 32) is then added to the bottom of part 30. There is a small hole and the alignment of part 32 is centered with the hole. Parts 44–49, the Person to Table Holders, are used to insert on top of the Table Top (part 17) to hold someone in place on their side when taking x-rays or making observations.

**Fig 23:**
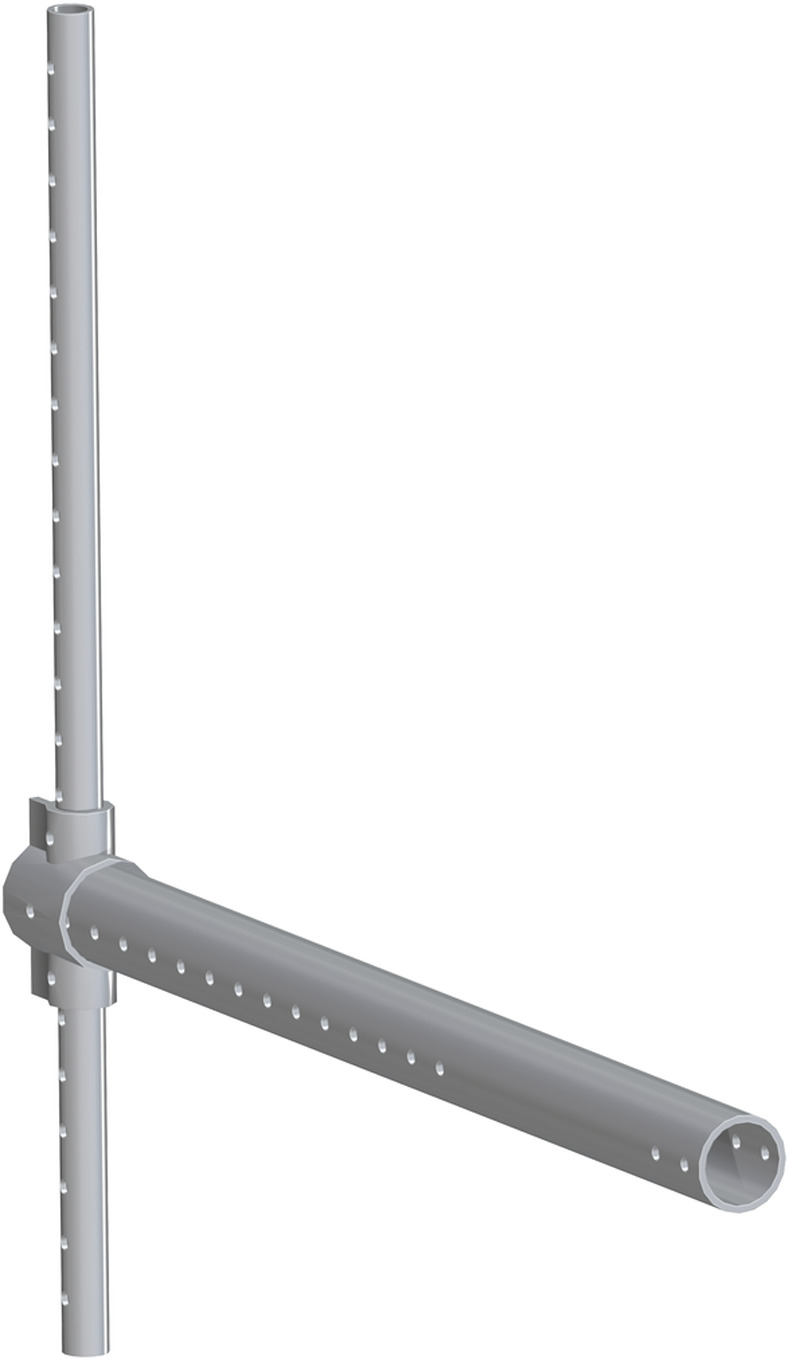
Insertion of 3in PVC to Leg vertical positioner.

*Single leg assembly*. The 1.25in pipe (part 33) is the base for this new assembly. The Leg vertical positioner (part 34) is then center aligned with part 33. The hole though the side of part 34 is center aligned with the eighth hole down on part 33. The 3in PVC (part 35) is center aligned with part 34. The end with more holes will be flush with the back side of part 34. There is a hole that will align with the holes from part 33 & 34. A hole will need to be inserted through the top of part 35 so it slides though part 33. This assembly is shown in Fig 23.

**Fig 24:**
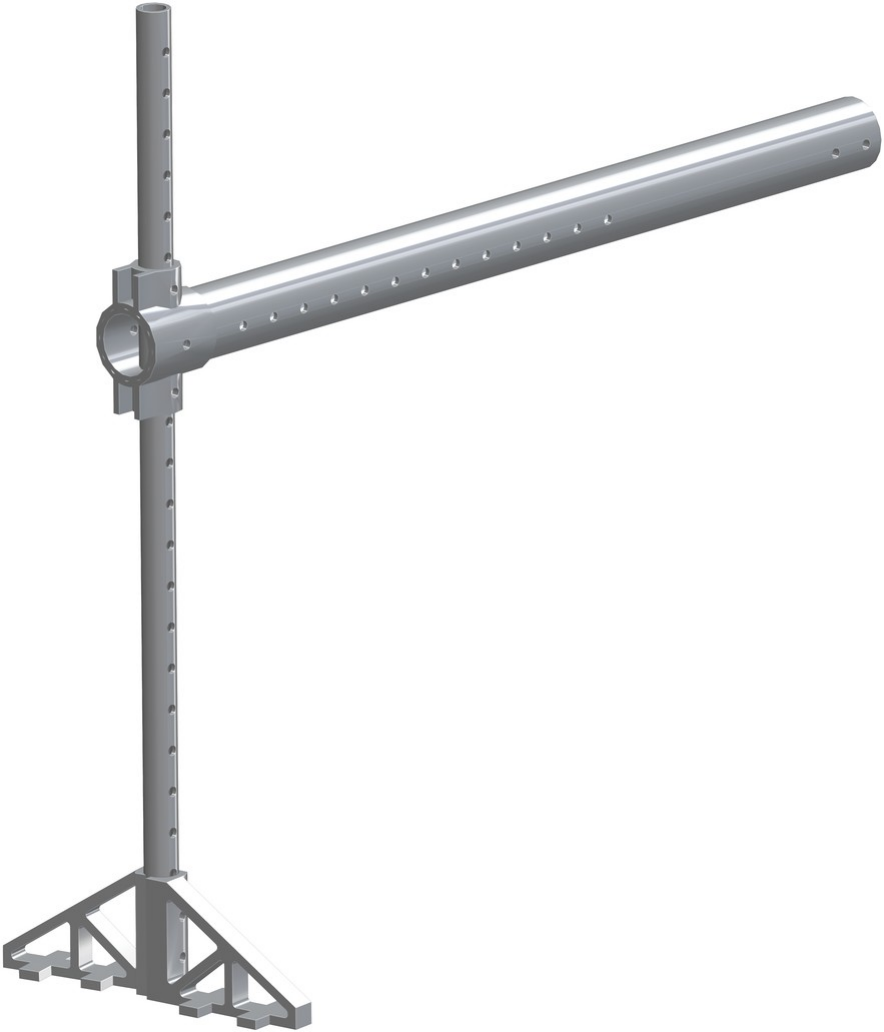
Addition of both vertical_pvc_leg_support_new to 1.25in pipe.

The vertical_pvc_leg_support_new (part 36) is to be added at the base of the 1.25in pipe. The bottom of part 36 is to be flush with the bottom of part 33. A second of part 36 will be added to the opposite side of part 33, to allow for support and stability. This is shown in Fig 24.

**Fig 25:**
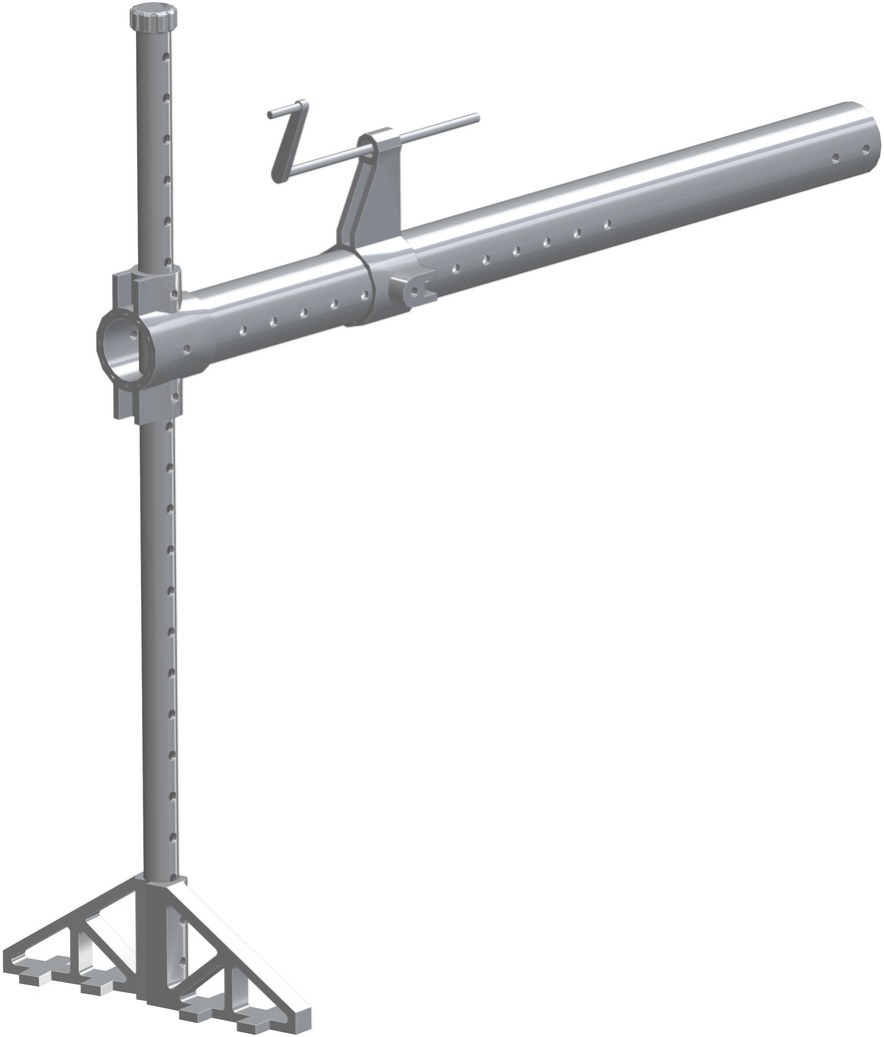
Addition of leadscrew_handle to the Lead Screw for Foot Pedestal.

The Foot Tensioner (part 37) is the next to be added to part 35. This is added along any of the side holes. In this particular assembly, it was added to the eighth hole from the end. It is center aligned with part 35. The Lead Screw for Foot Pedestal (part 38) is the next part to be added to part 37 in the top center hole. The top hole is threaded and will allow for the movement of part 38 to pass through. The pinched end faces towards the 1.25in pipe. The leadscrew_handle (part 39) is inserted on the pinched end of part 38. This allows for part 38 to be cranked in and out of part 37. This is shown in Fig 25.

**Fig 26:**
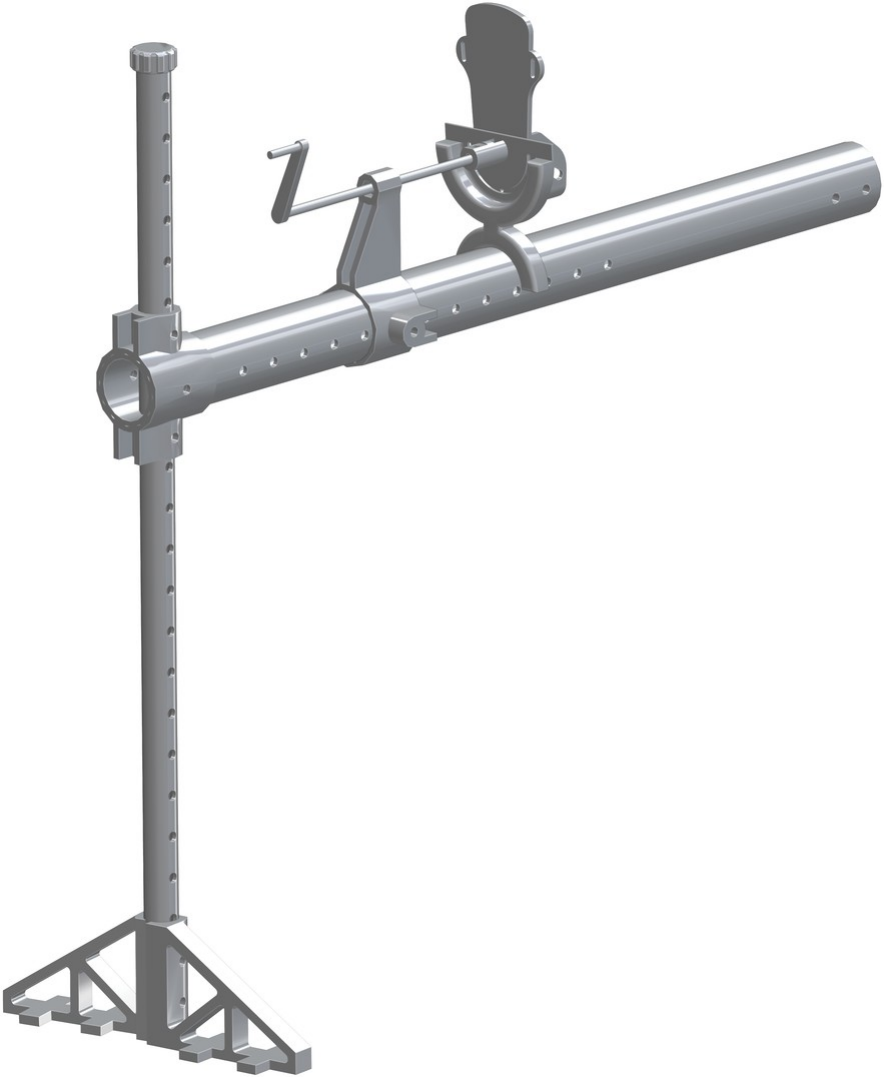
Addition of Foot-Rest to leg assembly.

The foot-rest_mount_with_angle_fix (part 40) is added to part 35, center aligned and on top. The hole on the backside is where the opposite end of part 38 inserts into. The Foot-Rest (part 41) is then added to the front side of part 40. There is a small hole where it screws into and is center aligned. The addition of part 41 completes the assembly of the single leg, shown in Fig 26. The Foot Fastener (part 52) can be attached to the lower hole in part 41 to enable it from free rotation.

Part 50–51, the Leg top holder, can be placed on top of the horizontal 3-in PVC (part 35) to allow for an extension of the table top.

**Fig 27:**
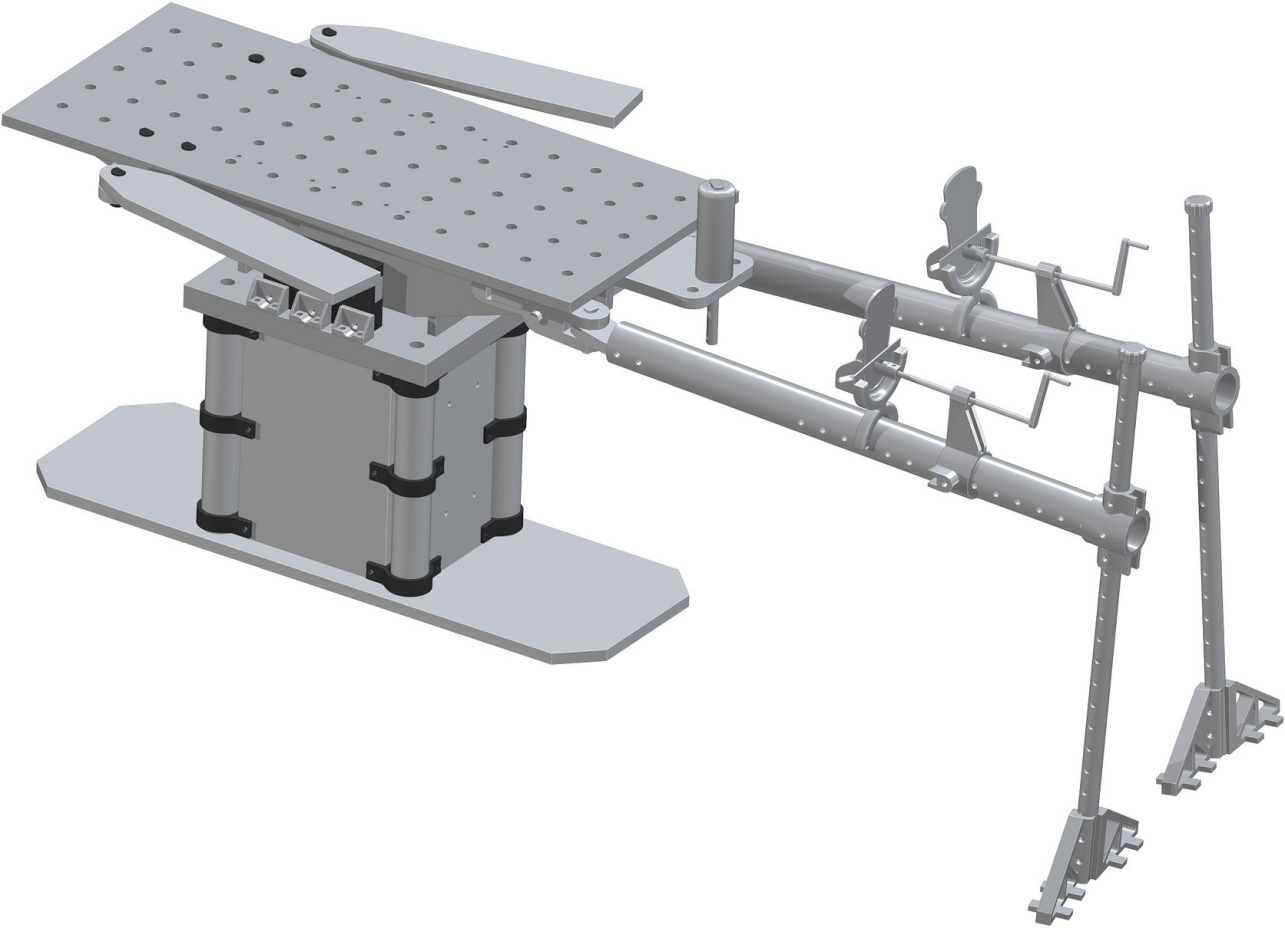
Final Table Assembly without cover and straps.

*Complete assembly of surgical table*. The complete surgical table is when both of the single leg assemblies are added after the peroneal post assembly. The Final Surgical Table Assembly is shown in Fig 27.

Finally, a cover is cut out of foam padding, of your choosing, to size (24x54 inches) and placed on the top of the table. This can be secured with clamps or velcro. Straps are placed on the foot holder and to the bottom of the table in order to secure the legs in position and minimize unintended movements.

### 2.3 Experimental testing and validation of mechanical design

The open source fracture table was fabricated as described in section 2.2 and then went through a number of tests to ensure that the mechanical actuation was as designed. The following mechanical movements were tested:

- Change in table height from minimum to maximum for each and both motorcycle jacks.
- Minimum and maximum table tilt at the highest height.
- Minimum and maximum height of legs
- Angle range of arm support.
- Angle range of leg support.
- Range of foot position.
- Angle of foot holder.

Finally, a stress test was performed on the table in the full up position to determine the mass the table could withstand using multiple researchers and finite element analysis was performed on the 3-D printed component under the greatest load.

## 3. Results

**Fig 28:**
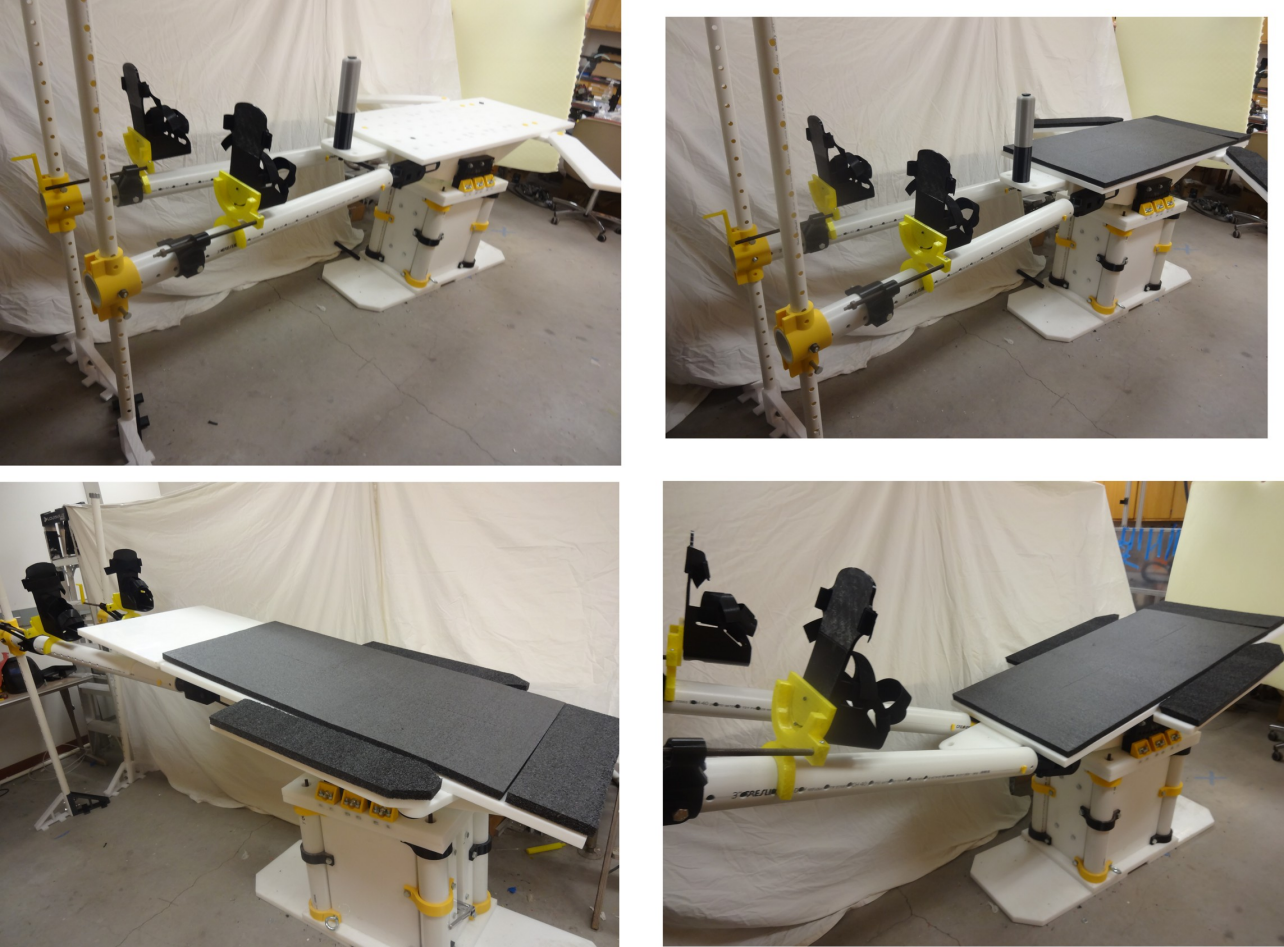
a) The final assembly with no pads. b) The final assembly with pads flat. c) The final assembly with pads tilted towards head (Trendelenburg position). d) The final assembly with the pads tilted towards feet (reverse Trendelenburg).

**Fig 29:**
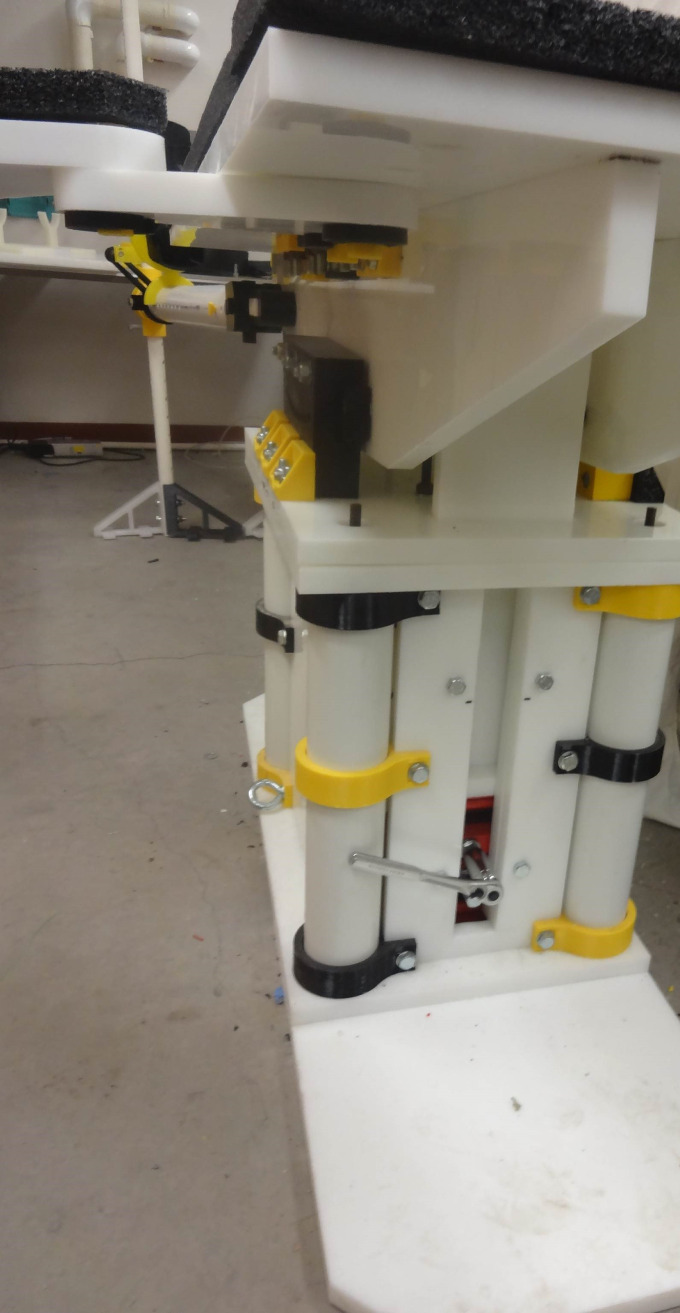
Details of wrench used to control heights of motor cycle jack and thus the table surface.

The open source fracture table was completely fabricated successfully as shown in Fig 28. Fig 28A) shows the fracture table without pads b) with pads, c) tilted toward the head and d) tilted toward the feet. Fig 29 shows the details of the wrench used to control extension of motor cycle jacks and thus the table surface height.

The results of the motion tests found that:

- Change in table height from minimum to maximum for each and both motorcycle jacks.
  - Minimum height was found to be 89.5 cm.
  - Maximum height was found to be 116.2 cm.
- Minimum and maximum table tilt at the highest height is +/-15 degrees.
- Minimum and maximum height of legs
  - The minimum height of the long PVC support for the legs was 30.5 cm.
  - The maximum height of the long PVC support for the legs was 116.8 cm.
- Angle range of arm support. The lateral motion of the arms was tested and found to move 180 degrees. The only item inhibiting it from moving any more is the table top.
- Angle range of leg support.
  - The lateral motion of the legs was found to be approximately 120 degrees.
  - The vertical motion of the legs was found to be 55 degrees.
- Range of foot position.
  - The position of the foot can be as close at 22 cm to the table end of the PVC pipe and as far as 76 cm, making for a range of 54 cm.
- Angle of foot holder.
  - The foot holder can angle 90 degrees to the left and to the right up the upright position.

**Fig 30:**
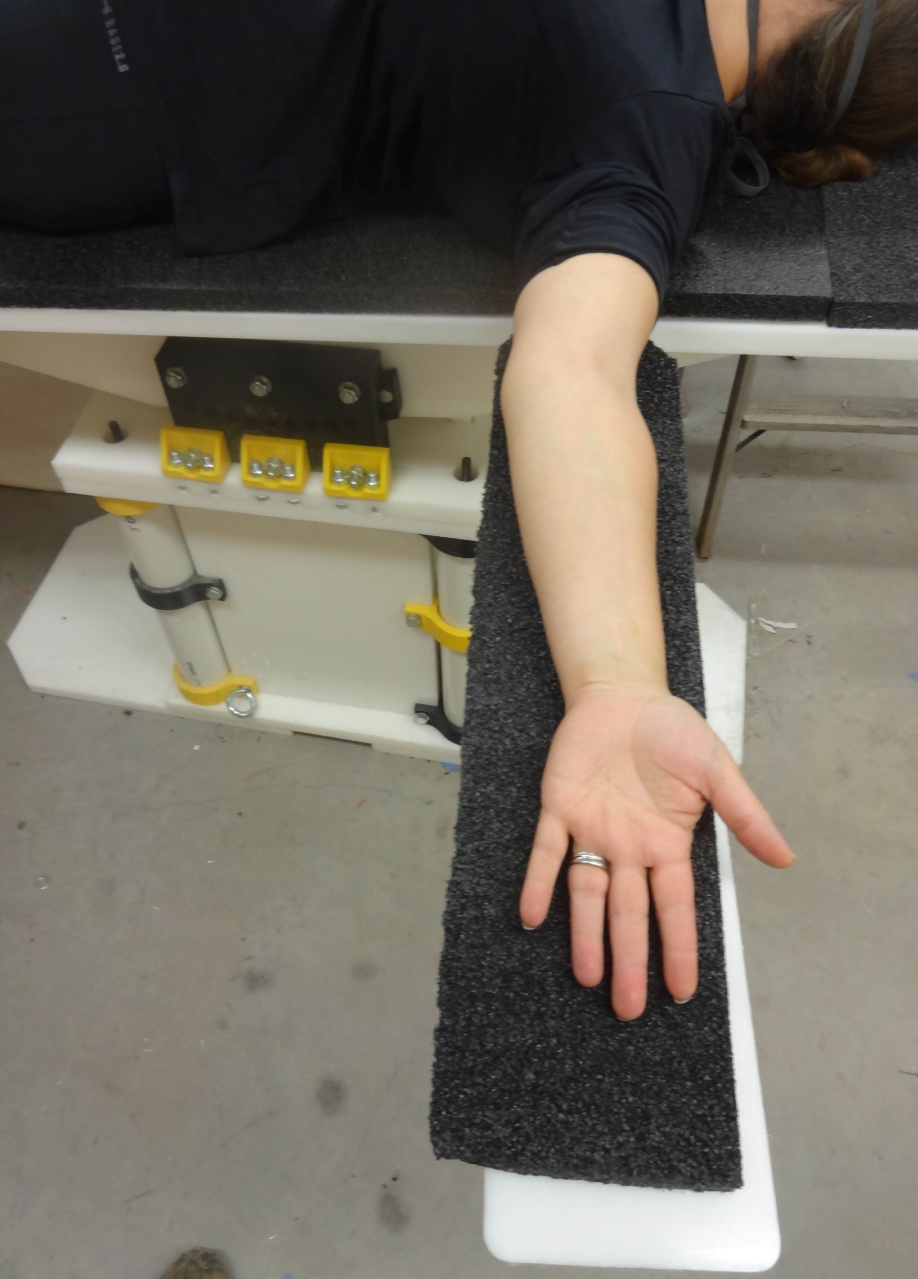
Details of arm support.

**Fig 31:**
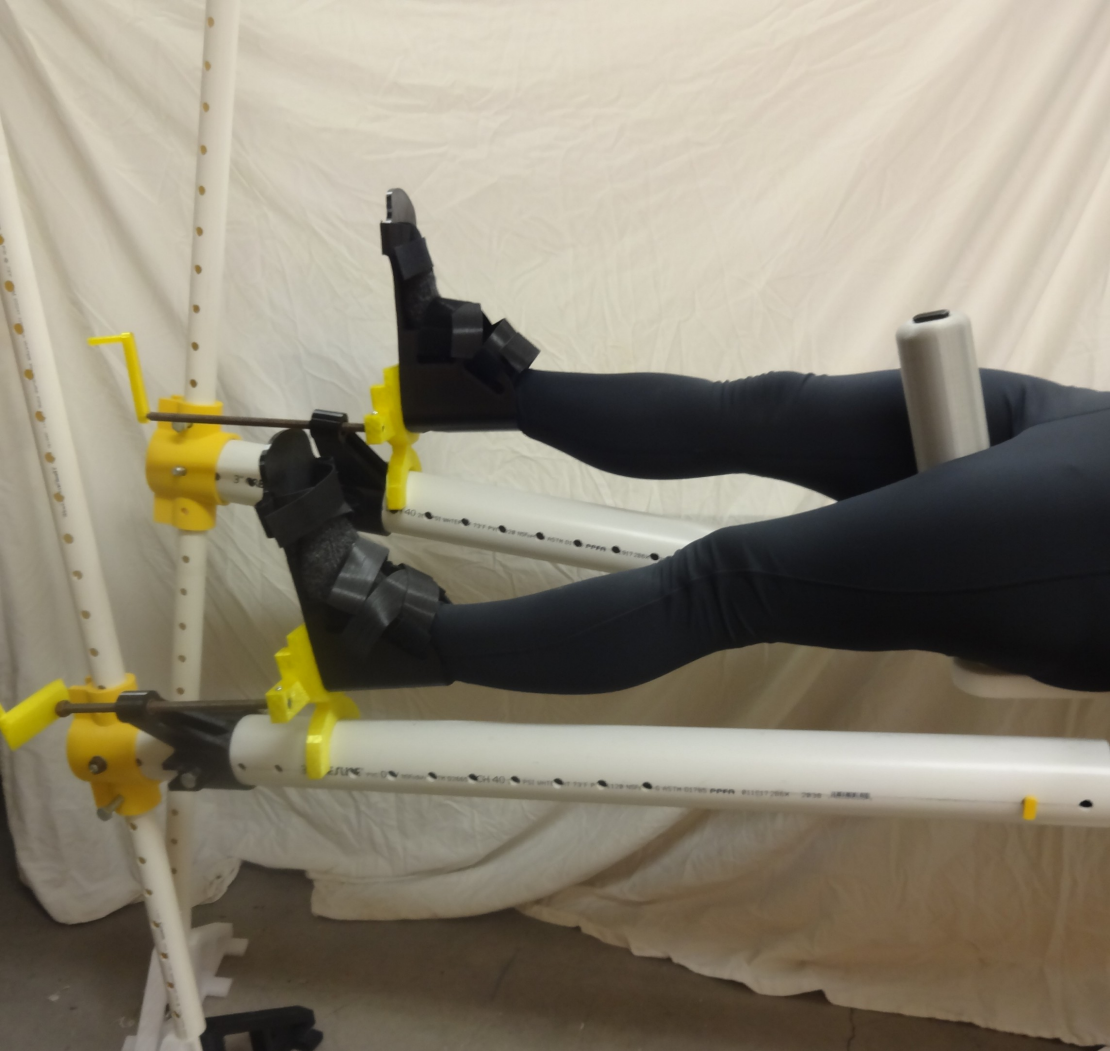
Details of one leg straight and one leg straight down (scissored).

**Fig 32:**
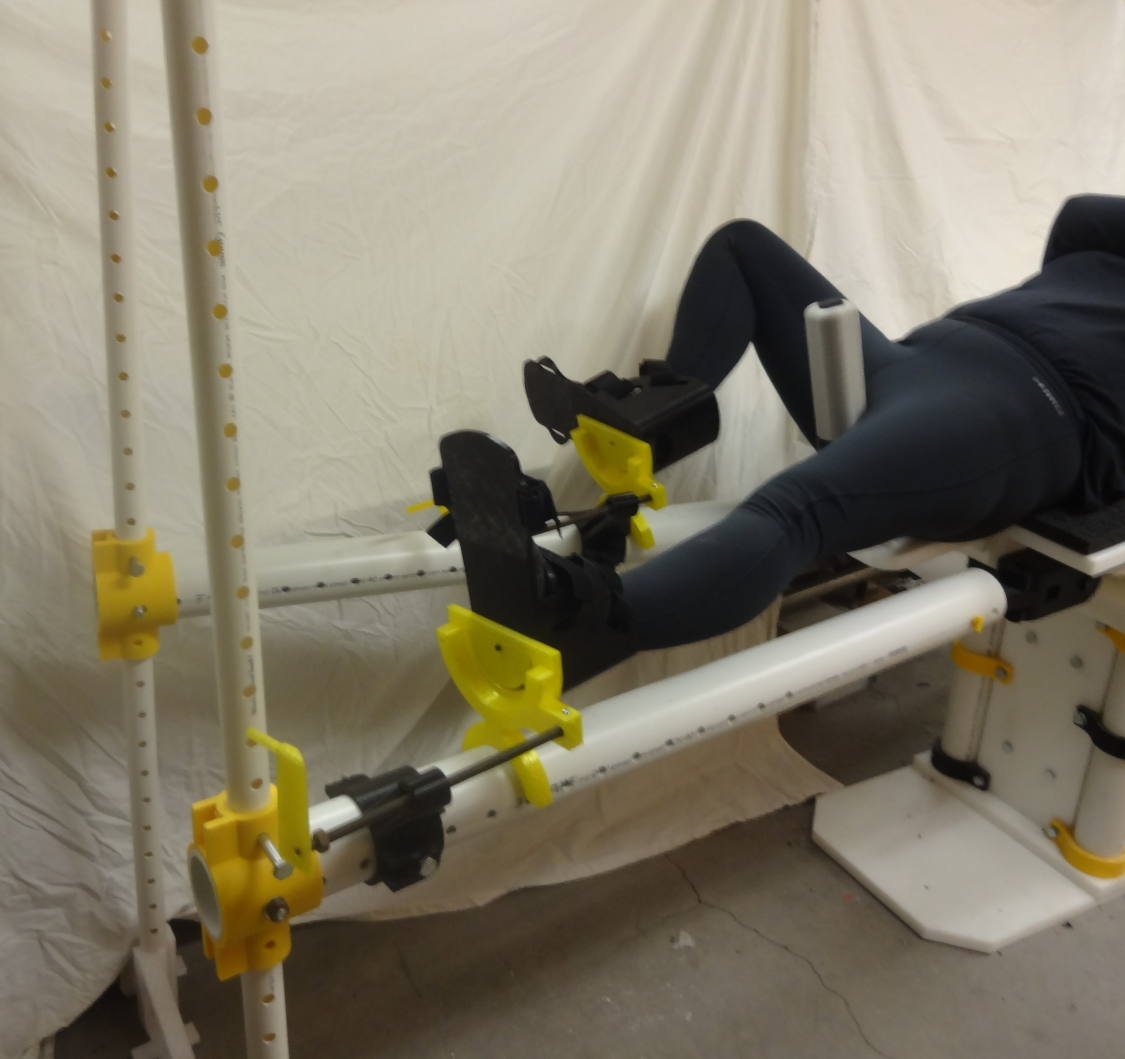
Details of one leg straight and the other flexed and abducted. Note that the leg positioner could have been abducted for this position.

**Fig 33:**
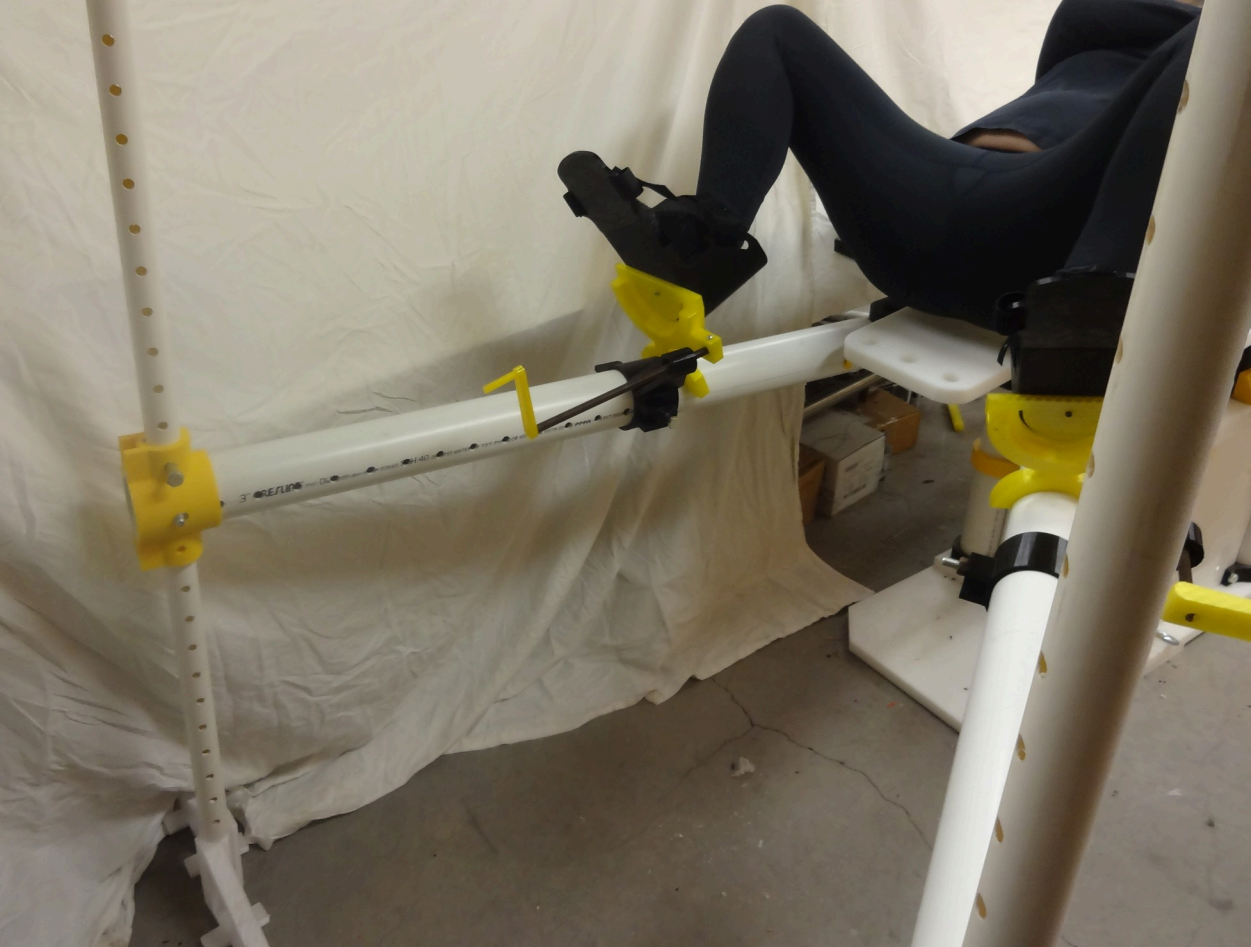
Table tilted down and both legs bent with no post.

**Fig 34:**
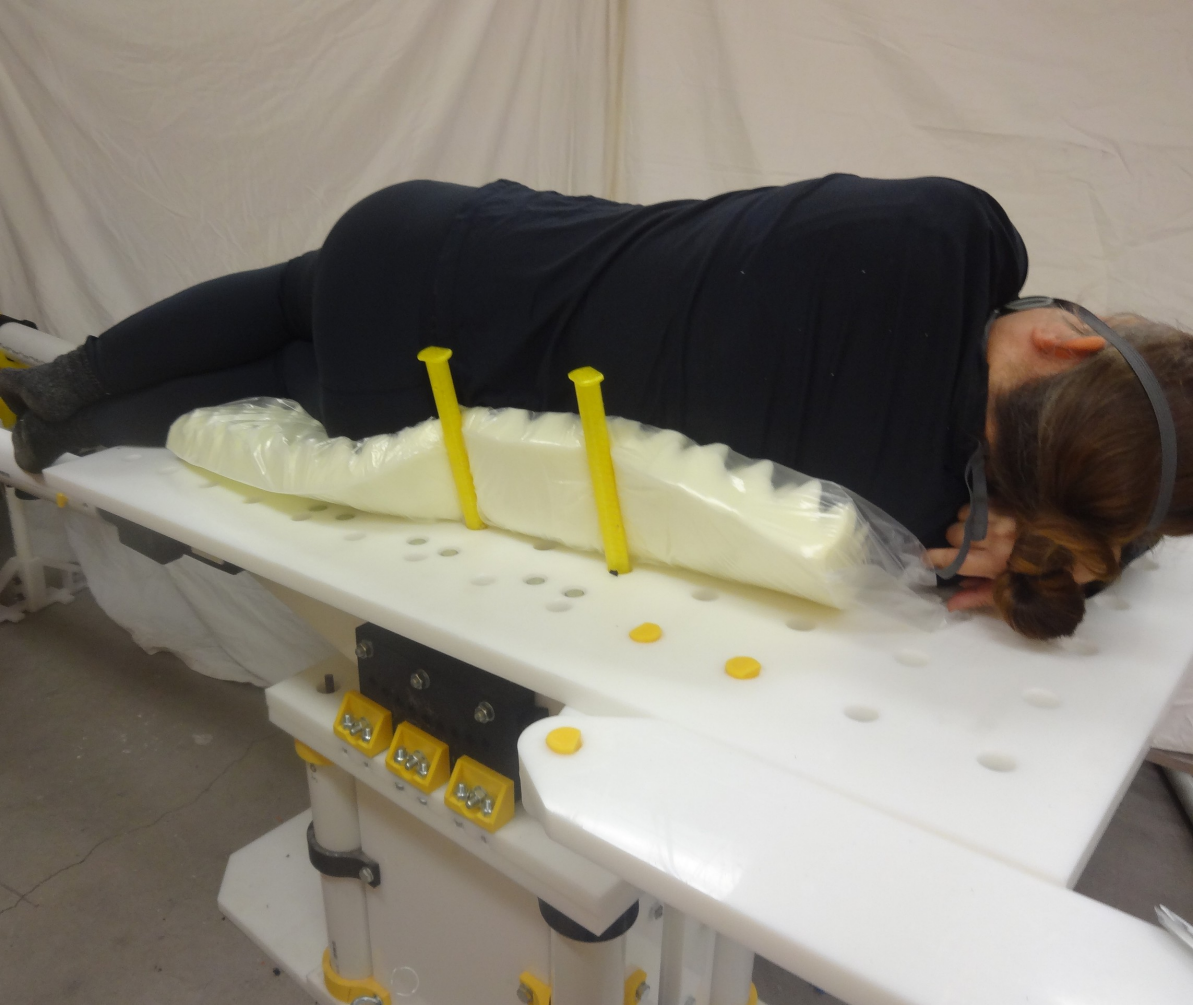
Patient on side.

The open source fracture table was demonstrated for five use cases shown in Figs 30–34. Fig 30 shows a simulated patient with their arm outstretched on the arm support when the table is flat for use to operate on an arm injury.

Fig 31 shows a simulated patient with their pelvis at the peroneal post and the feet strapped into the foot holders with one leg straight and one leg down (scissored).

Similarly, Fig 32 shows one leg straight while the other is held in an abducted and flexed position and this leg support moved up so the second leg is out of the way of the surgeon. Note that for this Figure, the leg positioner itself could be abducted much more to allow C-arm placement around a hip fracture.

Fig 33 shows the post removed with the table tilted down and the legs both flexed and abducted in order to help obstetrics and child birth.

Finally, Fig 34 shows a simulated patient resting on side for hip or shoulder surgery. Note the pegs are holding the patient in place.

**Fig 35:**
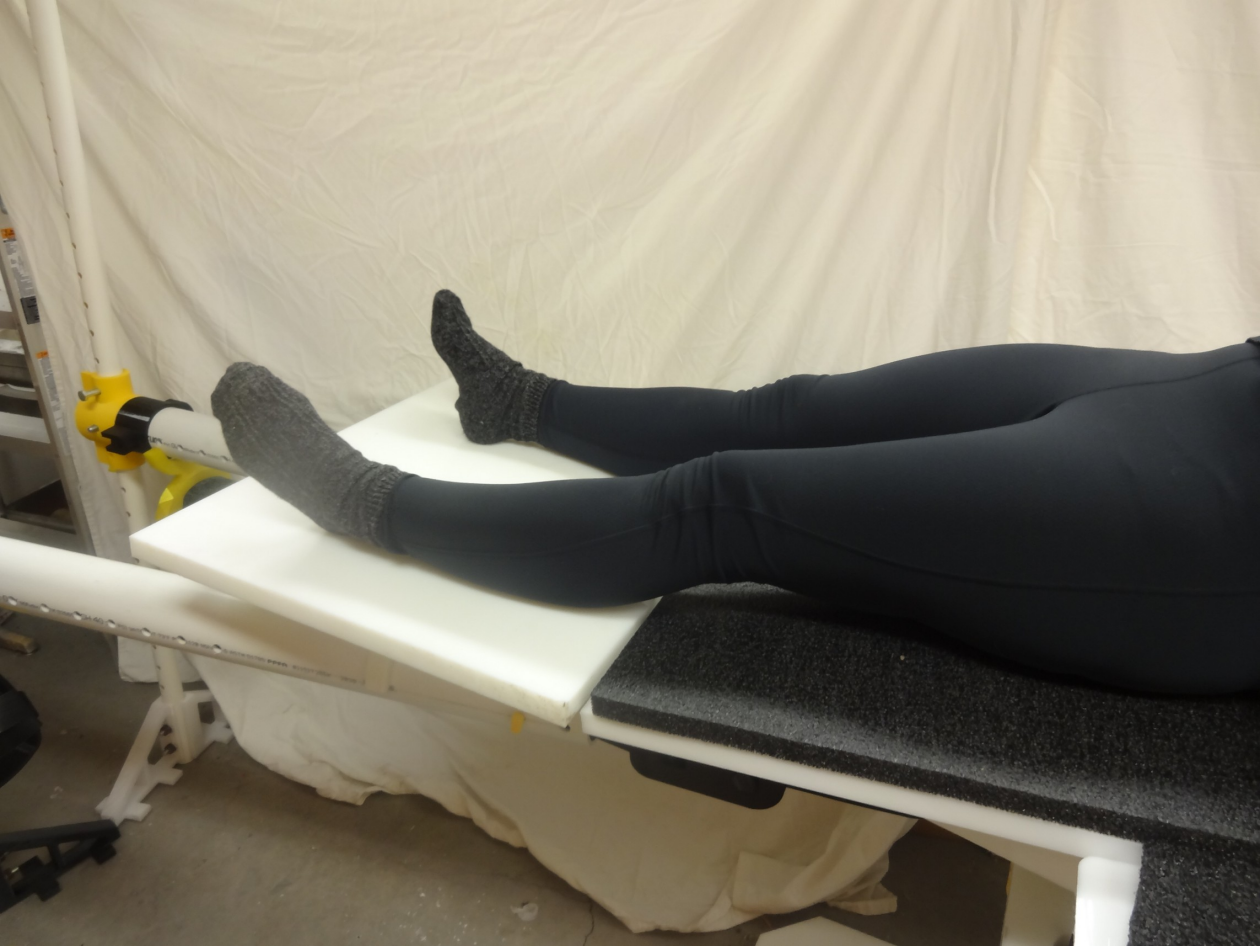
Table with simulated patient when table extension is inserted.

Fig 35 shows the table extension in place for foot, ankle, or leg surgery.

**Fig 36:**
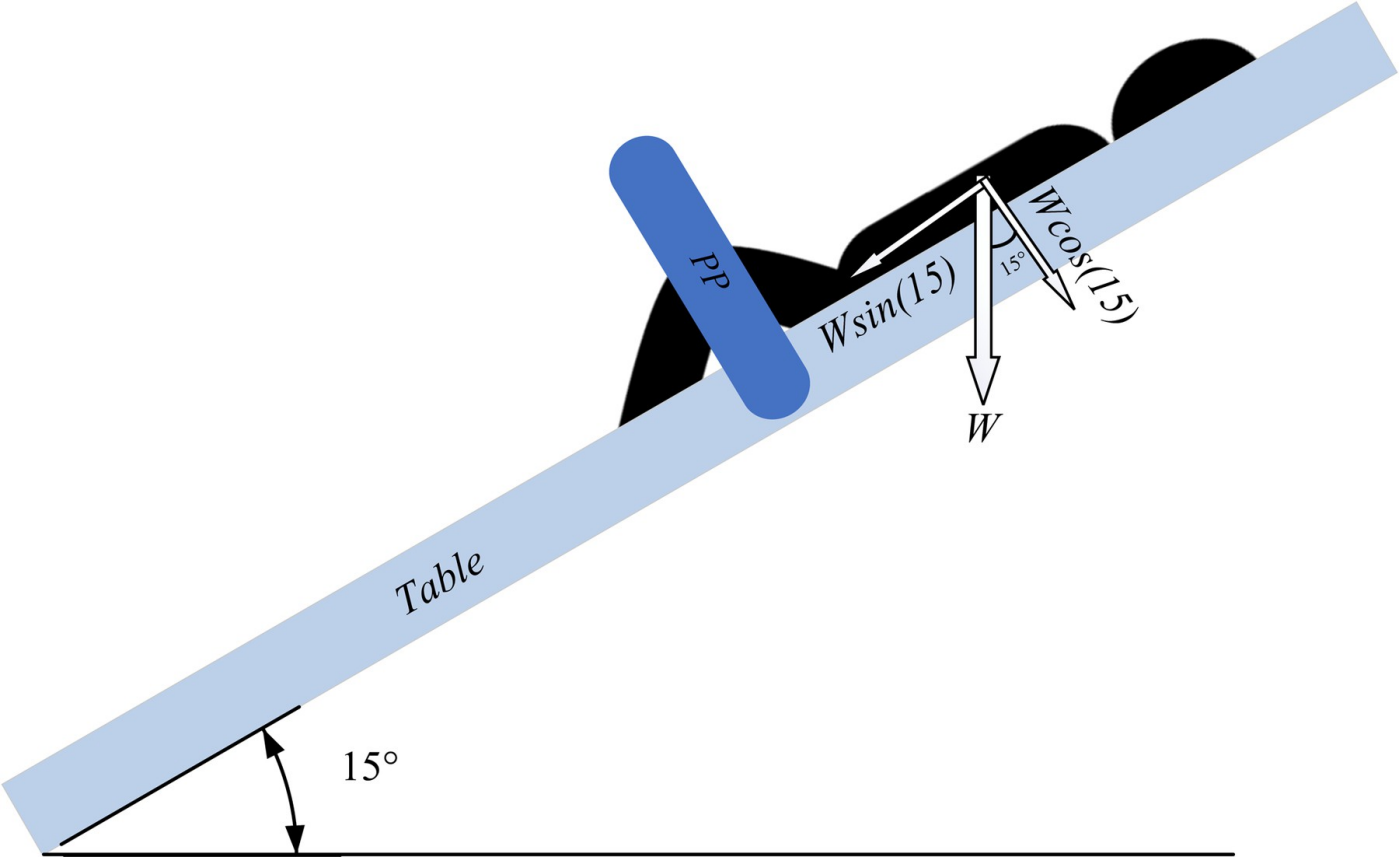
Simple diagram illustrating the applied force on PP in worst case scenario.

For the stress test, three researchers were asked to sit on the table in full-extension position. The total weight of stress test that the design was able to hold comfortably was 270 kilograms. This can be considered the upper bound of the mass of an individual that should use the table. The motorcycle jacks can easily accommodate the full 270 kg, however, depending on the material used for the peroneal post (PP), which is the component with the potentially highest force concentration, the upper limit of the apparatus can be lower. To calculate this a worst-case scenario was adopted where the patient would be laying on the table when it was adjusted tilted down to the maximum extent as shown in Fig 36, with the full weight of the patient on the 3-D printed PP.

The maximum weight (*W*) and mass (*M*) of the patient laying down on the fracture table under angle of 15 degree was calculated based on finite element method (FEM) analysis of the PP (diameter = 23 mm, length of under-load area = 393 mm) in *Abaqus*/*CAE*. Hexahedra mesh elements with a linear shape function-second order accuracy have been assigned to the objects, and no distorted elements have been reported by the software. As well, the size of elements (seeding and meshing) were decreased so that no difference on the output was observed.

The PP is made of PETG, therefore a sensitivity of the mechanical properties including the yield strength of it can be extracted from manufacturer specifications for the pure material [55], and sub-optimal 3-D printing PETG properties [56]. Furthermore, a ½ inch standard PVC pipe [57] (actual inside diameter = 15.3 mm, actual outside diameter:21.34, length = 393 mm) is considered as an alternative approach to the 3-D printed PP. The mechanical properties of PVC were extracted from [58]. The FEA results of a PP manufactured from 3-D printed PETG and replacing the 3-D printed component with an approximately equivalent diameter of PVC pipe are compared.

**Fig 37:**
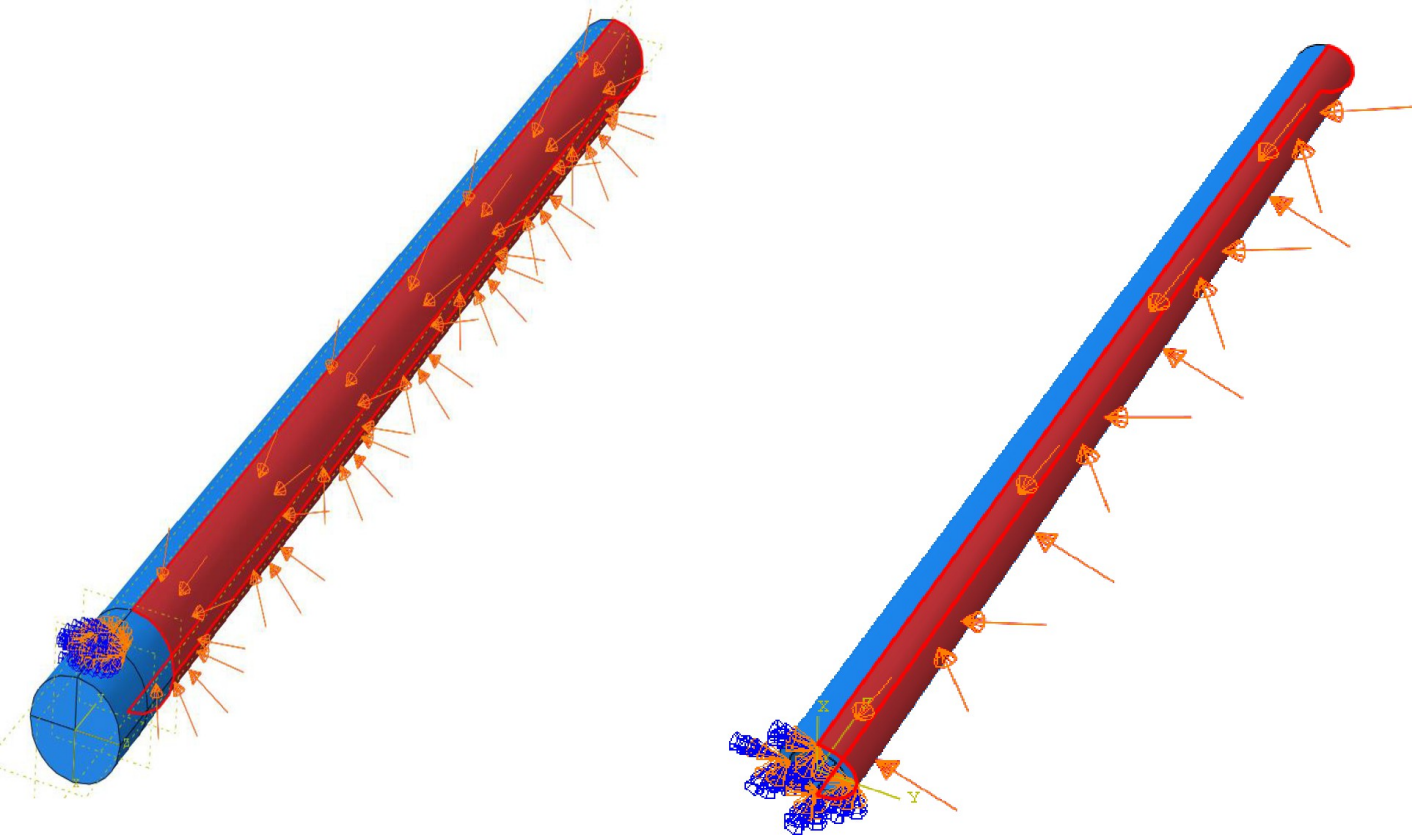
Pressure applied on the PP (left) and PVC pipe (right) in Abaqus.

In order to model the weight of patient on the PP that is 3-D manufactured and made from PVC pipe in *Abaqus*, it is assumed that a uniform pressure (*P*) has been applied on the half of their cylindrical area as Fig 37 indicated, then using Eq 1, *W* and *M* of patient can be calculated. Under pressure area can be calculated based on Eq 2. As previously stated, base angle is 15 degree, hence according to Fig 36, the effective force component in cracking PPand PVC pipe is *Wsin(15)*. Following the procedure mentioned in Eq 1, *W* and *M* can be calculated from the P = Force/Area using Fig 36.

$$\begin{array}{l} PPM\Rightarrow P=\frac{F}{A}=\frac{W\times \sin15^{\circ }}{0.014}=18.4871\times W\to W=0.054092P,W=9.81M\to M=0.005514P\\ PVC\Rightarrow P=\frac{F}{A}=\frac{W\times \sin15^{\circ }}{0.0132}=19.6075\times W\to W=0.051P,W=9.81M\to M=0.0052P \end{array}$$ (1)

$$\begin{array}{l} PPM\Rightarrow r=11.5mm,l=393.4mm,A=\frac{2\pi rl}{2}=0.014m^{2}\\ PVC\Rightarrow r_{out}=10.67mm,l=393,4,A=\frac{2\pi rl}{2}=0.0132m^{2} \end{array}$$ (2)

The pressure applied is shown in Fig 37.

**Fig 38:**
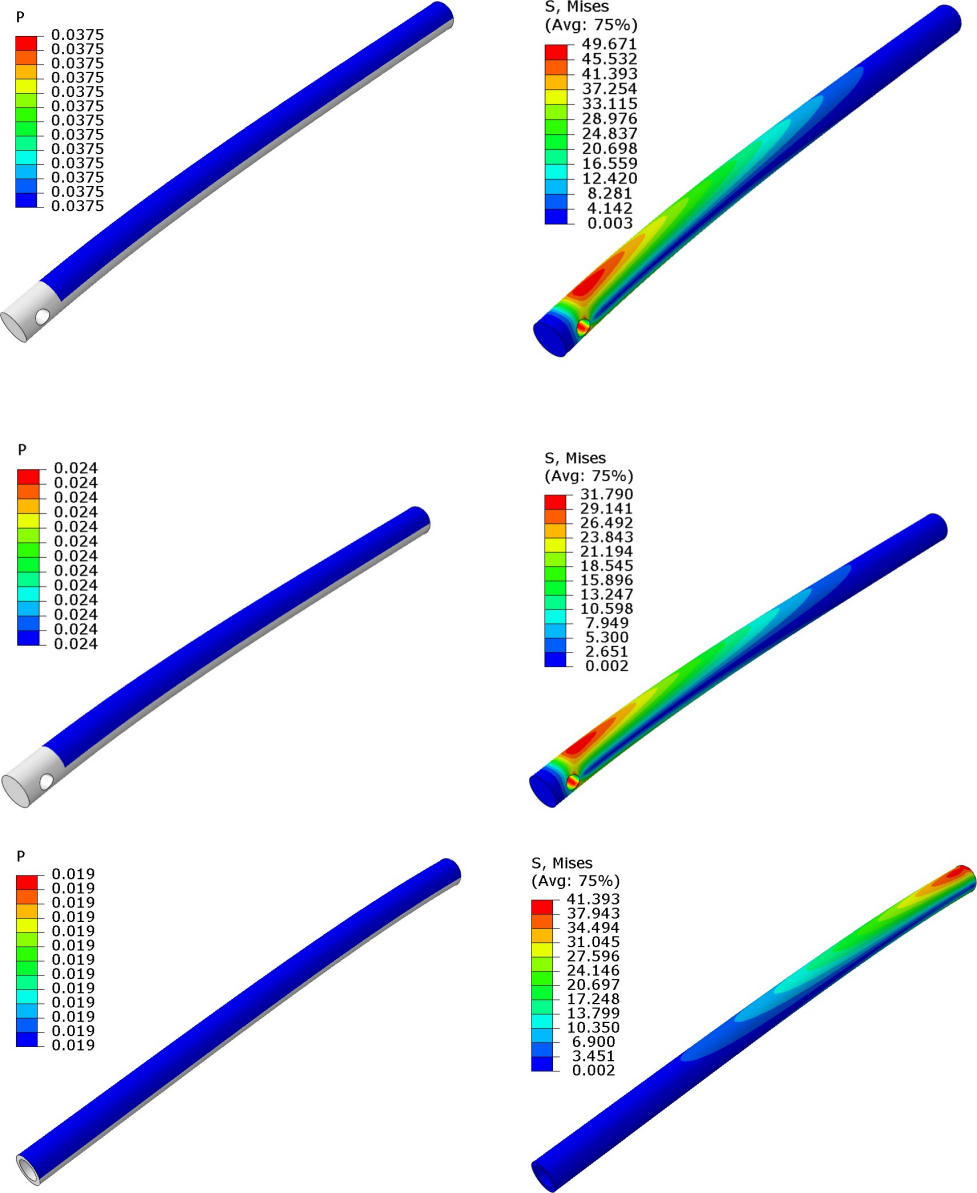
FEM analysis results for the maximum pressure (*P*), tolerable by a) PP for PETG manufacture’s specification, b) PP made with poor 3-D printing and c) PP with PVC pipe.

For calculating the maximum *W* and *M* of a patient that can utilize the open source surgical fracture table, when using a PP from 3-D printed PETG or PVC pipe can tolerate, several simulations have been conducted. In each simulation, maximum stress on PP and PVC pipe is monitored, while the pressure on PP was increased in simulations contentiously so that maximum stress matches the sensitivity of tensile strength of PETG [55,56]. The results are shown in Table 5A–5C). As a result, maximum *W* and *M* of patient, tolerable by PP and PVC pipe are illustrated with yellow color in the following table. Values indicated with red were passed from allowable yield strength of proposed materials and values showed with green are in the acceptable range. The details of the FEM analysis are shown in Fig 38 for the PP solid PETG, poorly 3-D printed PETG and PVC, in a, b, and c, respectively. It is clear from Table 5 and Fig 38, that the PP is actually the component that limits the maximum mass of a patient using the fracture table to 157 kg if using solid PETG and that when 3-D printed on a questionable quality 3-D printer this should be further reduced to 132 kg. To ensure that the 3-D printed PETG has the strength closer to that of the bulk material, it should be inspected for visual defects on the exterior surface and then massed to compare the mass of the component to its theoretical mass as discussed in [37]. To provide further confidence in the mechanical properties of the 3-D printed components, in-situ process monitoring can be used [59,60] of which several open source approaches [61] including those using computer vision and a two camera [62,63] and single camera are available [64,65]. Such continuous monitoring with a computer vision ensures that there is no under extrusion on the 100% infill in the 3-D print, that is difficult to detect in the final component.

**Table 5:** a. Effect of stress on PP with manufacture specifications. b. Effect of stress on PP with poor 3-D printing. c. Effect of stress on PVC pipe.

**A**			
**Pressure (MPa)**	**Maximum Stress on PP (MPa) based on manufacture specifications for PETG-Yield Strength = 50 MPa**	**W (N)**	**M (kg)**
0.0185	24.50	1000.7	102
0.019	25.17	1027.7	105
0.024	31.79	1298.2	132.3
0.025	33.11	1352.3	137.85
0.0285	37.75	1541.6	157.1
0.0375	49.67	2028.4	206.8
0.0385	51		
**B**			
**Pressure (MPa)**	**Maximum Stress on PP (MPa) based on poor PETG 3D printing-** **Yield Strength = 32.3 MPa**	**W (N)**	**M (kg)**
0.0185	24.50	1000.7	102
0.019	25.17	1027.7	105
0.024	31.79	1298.2	132
0.025	33.11		
0.0285	37.75		
0.0375	49.67		
0.0385	51		
**C**			
**Pressure (MPa)**	**Maximum Stress on PVC pipe (MPa)-** **Yield Strength = 41.4 MPa**	**W (N)**	**M (kg)**
0.0185	40.3	943.5	96.2
0.019	41.39	969	98.8
0.024	52.29		
0.025	54.46		
0.0285	62.09		
0.0375	81.70		
0.0385	83.87		

If the open source surgical fracture table needs to be used for heavier patients than 130 kg then the PP could be manufactured using a high strength polymer such as polycarbonate on a conventional FFF 3-D printer or using a high-temperature FFF 3-D printer an engineering polymer commercially available as filament like polyetherketoneketone (PEKK) and polyetherimide (PEI, ULTEM) with tensile strengths of 77.5 and 80.5 MPa, respectively [19]. It is not recommended to use PVC pipe as the PP as this limits the mass of the patient to 98 kg as seen in Table 5C.

## 4. Discussion

Orthopedic injuries and disease continue to be a significant cause of death and disability worldwide. Low- and middle-income countries, in particular, suffer from a lack of resources and personnel to provide quality and effective care to deal with many commonly seen diagnoses. For surgical care, equipment, instruments, implants, pharmaceuticals, dressings, splints or casts, rehabilitation equipment and trained personnel are all necessary for the patient to have an optimal outcome. When resources are scarce, first-line treatment may not be universally available, or available at all. Many strategies have been applied to improve the care and outcomes of patients in under-resourced areas. Some of these strategies have included donating used and outdated equipment from better-off regions, but this strategy often fails when the equipment fails and cannot be repaired, when the local personnel are not trained in the use of the equipment, or when the technology is obsolete and there is no use or support for it. Worse, many of these donated items end up becoming a burden to the hospitals that receive them as there is little room for storage and upkeep of the equipment. When procuring equipment for under-resourced areas, it is crucial that the equipment be able to be understood by the local users and be able to be maintained locally. The design of this surgical table uses all easily-sourced and 3-D printable pieces in order to be able to be fixed and maintained, wherever it is being used. In addition, by being built and assembled locally, the local users should know and understand the design, thus being able to service the table as necessary. In addition, it should be pointed out that the designs, although radically less costly than current new models, could still be made much lower cost following the tenants of the circular economy applied to 3-D printing [66,67] using distributed recycling and additive manufacturing (DRAM) [68,69]. The 3-D printed components make up about 10% of the cost, which could be virtually eliminated [70] using recycled filament from a recyclebot [71,72] and a similar RepRap-class 3-D printer [73] to the one used, or direct fused particle fabrication [74]. In addition, a Precious Plastic [75] or similar open source hot press [76] could be used to make recycled HDPE sheet blocks, which are roughly 2/3rds of the total cost of the open source table. Combining these two approaches would result in a surgical fracture table that would be accessible in most contexts.

There is a lack of qualified doctors and surgeons in many countries, and many under-resourced areas suffer from a “brain-drain” of their medical personnel who travel to be trained in larger and better resourced centers and then do not return to an area that has less resources. As well, the training of physicians overseas may not provide applicable education to these physicians as the problems that they see and the technologies that are available to them in their countries of origin are completely different than what is available in developed nations. Diseases such as Human Immunodeficiency Virus, Acquired Immune Deficiency Syndrome and Tuberculosis are much more prevalent in the developing world and cultural perspectives on disease and dying are often quite different [8]. Thus, trainees returning to their home countries may find their training, while technically excellent, may have deficiencies when it comes to their treating the local population [77]. They may find as well that the methods that they were trained in cannot be performed with the resources that are available to them at home, causing further alienation of the medical providers. This is one of the reasons that it is important to have modern features available for equipment designed for under-resourced areas, so that the features can provide what is needed by the medical staff, and be similar to what they have trained with. In this way, the procedures are familiar and easily-reproducible. This is also why it is so important for procedures to be designed with limited technology (like the SIGN) and medical providers in under-resourced areas trained specifically in these procedures, so that patients are able to be treated locally with effective and safe procedures using technologies that are locally-available. This table design has attempted to capture most modern fracture table functionalities in a mechanical design that does not rely on electricity. It is also modular to allow easy adjustments and improvements to the design at the local level to solve local issues.

Like any medical technology, it is important that new designs be rigorously tested in a non-patient care setting prior to using them with patients who are vulnerable and at the mercy of their medical providers. When the table is built locally, the strength and stability of the table and leg supports needs to be tested before use. This table should only be used by qualified medical personnel. All personnel involved in procedures using the table should be trained on the use of the table, the functionality of the application of traction, Trendelenburg and reverse Trendelenburg positioning, modifications of the heights of the table itself, as well as of each of the legs independently, and securing the arm supports and leg supports. The peroneal post must always be in position and locked when traction is applied. The table needs to be padded, the peroneal post must be padded and all bony prominences on the patient should be padded for any surgical procedures as the patient cannot notify the team of discomfort if there is a pressure point under anaesthesia, which could result in nerve injury, ischemia and necrosis. Traction should only be administered under direct medical supervision and for the minimum time that it takes to position and secure the fracture. Finally, future work is needed to test the lifetime of the components and the design used in a clinical setting. All use of the table and its designs are at the risk of the user.

Ideally this table design is useful to hospitals and clinics that might otherwise be unable to afford such technology and that, as the design of the table is freely available, the table will be able to be built, used and repaired on location. As physicians, surgeons and other health care workers find a need for further attachments and modifications, they can add to the functionality of the design, either by designing a modification themselves, or by describing what they would like to see in the design and having others contribute to these designs. By having modern functional equipment available locally and being able to be serviced locally, access to quality musculoskeletal care should improve.

## 5. Conclusions

This paper successfully describes the design of an open source surgical fracture table, which can be manufactured locally with common hand tools and a desktop 3-D printer. The open source fracture table costs approximately $3,000 USD in materials and has comparable features to commercial proprietary systems that cost over $200,000. It has verified performance for mechanical loading, geometric flexibility to allow for wide array of common surgeries, is radiolucent in surgical zones, and is modular and upgradeable.
